# Hyperalgesia and Persistent Pain after Breast Cancer Surgery: A Prospective Randomized Controlled Trial with Perioperative COX-2 Inhibition

**DOI:** 10.1371/journal.pone.0166601

**Published:** 2016-12-09

**Authors:** Noud van Helmond, Monique A. Steegers, Gertie P. Filippini-de Moor, Kris C. Vissers, Oliver H. Wilder-Smith

**Affiliations:** 1 Department of Anaesthesiology, Pain and Palliative Medicine, Radboud University Nijmegen Medical Centre, Nijmegen, The Netherlands; 2 Department of Anesthesiology, Bernhoven Hospital, Uden, The Netherlands; 3 Center for Sensory-Motor Interaction (SMI), Department of Health Science and Technology, Aalborg University, Aalborg, Denmark; Maastricht University Medical Centre, NETHERLANDS

## Abstract

**Background:**

Persistent pain is a challenging clinical problem after breast cancer treatment. After surgery, inflammatory pain and nociceptive input from nerve injury induce central sensitization which may play a role in the genesis of persistent pain. Using quantitative sensory testing, we tested the hypothesis that adding COX-2 inhibition to standard treatment reduces hyperalgesia after breast cancer surgery. A secondary hypothesis was that patients developing persistent pain would exhibit more postoperative hyperalgesia.

**Methods:**

138 women scheduled for lumpectomy/mastectomy under general anesthesia with paravertebral block were randomized to COX-2 inhibition (2x40mg parecoxib on day of surgery, thereafter 2x200mg celecoxib/day until day five) or placebo. Preoperatively and 1, 5, 15 days and 1, 3, 6, 12 months postoperatively, we determined electric and pressure pain tolerance thresholds in dermatomes C6/T4/L1 and a 100mm VAS score for pain. We calculated the sum of pain tolerance thresholds and analyzed change in these versus preoperatively using mixed models analysis with factor medication. To assess hyperalgesia in persistent pain patients we performed an additional analysis on patients reporting VAS>30 at 12 months.

**Results:**

48 COX-2 inhibition and 46 placebo patients were analyzed in a modified intention to treat analysis. Contrary to our primary hypothesis, change in the sum of tolerance thresholds in the COX-2 inhibition group was not different versus placebo. COX-2 inhibition had an effect on pain on movement at postoperative day 5 (p<0.01). Consistent with our secondary hypothesis, change in sum of pressure pain tolerance thresholds in 11 patients that developed persistent pain was negative versus patients without pain (p<0.01) from day 5 to 1 year postoperatively.

**Conclusions:**

Perioperative COX-2 inhibition has limited value in preventing sensitization and persistent pain after breast cancer surgery. Central sensitization may play a role in the genesis of persistent postsurgical pain.

## Introduction

Persistent pain after surgery is a significant clinical problem which affects 10 to 50 percent of patients [1]. Chronic pain treatments are effective in reducing pain in only about 30 percent of patients with such persistent pain [2]. In breast cancer surgery similar outcomes are reported, with around 40 percent of patients suffering from persistent pain one year after surgery [3, 4]. These results are not surprising in view of the complexity of persistent pain and current empirical symptom-based pain management approaches. Further improvement in persistent and chronic pain management will likely depend on the development of more mechanism-based approaches [5, 6].

A key insight from fundamental pain research is that ongoing nociceptive input alters subsequent sensory processing by the nervous system [7]. Surgical nociception results in postoperative hyperalgesia via pronociceptive changes in central nervous system processing. Such ‘central sensitization’ occurs via two mechanisms, namely damage to tissues and to nerves, with the former acting more via humoral biochemical products of tissue inflammation, and the latter more via neuronal mechanisms [7]. Postoperative central sensitization and hyperalgesia not only lead to increased acute pain, they have also been linked to subsequent development of chronic pain [8–13]. Preventing postoperative central sensitization may therefore provide an attractive mechanism based approach to prevent persistent pain development, e.g. by blocking nociceptive input or direct antihyperalgesic therapy [14–18].

Regional anesthesia is currently the best therapy to block surgical nociceptive input and may protect partially against persistent pain development after surgery [19–21]. However, even with paravertebral block around twenty-two percent of women undergoing breast cancer surgery suffer from persistent pain six months after surgery [22, 23]. To further improve management of surgical pain it would be useful to understand the effect of adding inhibition of the inflammatory component of sensitization, e.g. by providing perioperative cyclooxygenase-2 (COX-2) inhibition [24–26] in addition to blockade of neuronal nociceptive input. COX-2 inhibitors interfere with prostaglandin production [27] and may counteract central sensitization development by inhibiting peripheral sensitization [27] and reducing nociceptive input. Additionally, COX-2 inhibitors may prevent central sensitization by a central mechanism [24, 27].

The primary aim of this study was to assess the value of perioperatively inhibiting the inflammatory component of sensitization added to block of neuronal nociceptive input on central sensitization after surgery. A secondary aim was to assess the relationship between hyperalgesia and persistent pain development at 12 months postoperatively. We studied these aims in a randomized prospective controlled trial in women undergoing breast cancer surgery under paravertebral blockade combined with perioperative COX-2 inhibition or placebo. We hypothesized that:

1. Adding COX-2 inhibition to standard maximal antinociceptive treatment (paravertabral blockade) perioperatively would result in less widespread hyperalgesia as a sign of central sensitization–and therefore less persistent pain–following surgery compared to a placebo-supplemented group.
2. Patients who complained of persistent pain 12 months postoperatively would exhibit more widespread hyperalgesia following surgery, than patients not complaining of persisting pain.

## Materials and Methods

We conducted a prospective, randomized, double blind, placebo-controlled, clinical trial at the Bernhoven Hospital in Uden, the Netherlands, approved by the Ethical Committee on March 16th 2005 (nr: 2004/239, CMO region Arnhem-Nijmegen, Nijmegen, The Netherlands). All participants provided written informed consent; the trial was registered with the Netherlands Trial Register (NTR1793). Trial registration was not complete when subject recruitment had begun. However, this was rectified and our trial was registered on May 3rd 2009. The authors confirm that all ongoing and related trials for this drug/intervention are registered. The protocol for this trial and supporting CONSORT checklist are available as supporting information; see S1 Checklist and S1 Protocol.

### Patients

We included women scheduled for breast cancer surgery. Two dedicated breast surgeons performed all surgeries. Surgery was by lumpectomy, total simple mastectomy or modified radical mastectomy. Exclusion criteria were: previous breast surgery, planned immediate breast reconstruction, chronic pain syndromes (e.g. fibromyalgia, osteoarthritis), regular analgesic medication for 2 weeks preceding surgery, pre-existing central nervous system pathology (e.g. stroke, dementia), conditions predisposing to neuropathy (e.g. diabetes mellitus, alcohol abuse), inability to comply with testing procedures or to give informed consent, presence of contra-indications to COX-2 therapy (including untreated hypertension, active or recent gastrointestinal ulceration) and contraindications to paravertebral blockade.

### Randomization and treatment

After obtaining informed consent during an outpatient anesthesia visit, eligible patients were randomized in a one-to-one ratio to receive perioperative COX-2 inhibition or placebo. A pseudo-random code was computer generated for the randomization blocks that had a size of six. Stratified random sampling ensured equal distribution of axillary lymph node dissections over groups. The hospital pharmacy held the randomization scheme for the trial and supplied parecoxib and celecoxib (active treatment) or placebo in blinded packages. Parecoxib is currently not FDA approved, but is widely available worldwide, including in the European Union as the only injectable COX-2 specific inhibitor. The morning of surgery, patients received oral midazolam premedication (7.5 mg). In the operating theatre, COX-2 inhibition group patients received parecoxib 40 mg i.v. 30 minutes before surgery start. This injection was repeated 6 hours later. The postoperative morning, patients started celecoxib 200mg, continued to the morning of day five postoperatively. The placebo group received placebo injections and tablets according to the same regime. Medication was blinded, neither observers nor persons involved in patient management were aware of patient assignment.

### Anesthesia and analgesia

Paravertebral blockade was by standard technique (20 ml ropivacaine 0.75%). Before surgery, local anaesthetic blockade was tested using pin-prick. Unsuccessful block, as defined by no hypoalgesia to pinprick, led to patient exclusion. Patients received standardized general anaesthesia [28], (propofol 2–3 mg/kg, fentanyl 3 μg/kg, rocuronium 0.5 mg/kg, air/oxygen (40%), sevoflurane) to achieve haemodynamic values within 20% of preoperative baseline. For procedures longer than 45 minutes, further fentanyl supplementation (1 μg/kg) was permitted at 45 minutes and at further 45-minute intervals. No further myorelaxants were given and no antagonisation was performed. In the recovery room, initial analgesia consisted of piritramide as soon as patients complained of pain, titrated to VAS≤3 by the recovery room nurse using 3 mg intravenous increments. Thereafter, standard postoperative analgesia consisted of a fixed acetaminophen scheme (4 X 1g /day) together with on-demand tramadol (drops, maximum 300 mg/day) up to day 5 postoperatively.

### Measurement protocols

Trained research personnel performed all testing in a standardized fashion in a quiet room. All subjects underwent familiarization training with sensory testing before the study. Pain was assessed via 100 mm visual analogue scores at rest (lying quietly in bed) and on movement (immediately after sitting up on bed). For all postoperative pain scores, the patient was explicitly asked to report pain associated with surgery at that moment. At several time points patients were asked to complete a quality of life questionnaire assessing surgery-related symptoms and functional impairment.

Postoperative changes in pain sensitivity (hyperalgesia) were quantified using electric and pressure pain tolerance thresholds. Electricity stimulates mainly cutaneous nerve endings [29], bypassing nociceptors; pressure reveals deep tissue sensitivity (e.g. muscle), with only minimal cutaneous contributions [30]. Thus electric pain tolerance thresholds mainly reflect cutaneous sensitivity and pressure pain tolerance thresholds mainly reflect deep tissue sensitivity. Thresholds were measured close to the affected breast and distant from the site of surgery to obtain measures of secondary (peri-incisional) and spreading (or generalizing) hyperalgesia, respectively. Pain modulation was assessed preoperatively via conditioned pain modulation (CPM) paradigm [31]. At no time were patients or treating personnel aware of results of pain processing tests.

Baseline demographic data, electric pain tolerance thresholds, pressure pain tolerance thresholds and CPM were collected the preoperative afternoon. Pain scores, electric pain tolerance thresholds and pressure pain tolerance thresholds were collected 1, 5 and 15 days after surgery and at 1, 3, 6 and 12 months after surgery.

### Electric and pressure pain tolerance thresholds

Electric pain tolerance threshold testing was performed using an electric stimulation device (QST-3; JNI, Aalborg, Denmark), delivering electrical tetanic stimulation (100 Hz, 0.2-ms square waves, 0.1mA/s ramping rate) via self-adhesive skin electrodes 3 cm apart. A trained research assistant operated the device and documented the value at which stimulation became intolerable and was discontinued. Pain tolerance thresholds were determined three times and the mean value was used. Pressure pain tolerance thresholds were assessed using a pressure algometer (Somedic Sales AB, Horby, Sweden) with a 1.0 cm2 probe and a ramping rate of 50 kPa/s[28] until the patient did not accept a higher stimulus intensity. The electric pain tolerance thresholds were measured at each of the following sites on both the affected body side and the contralateral side: Radial upper arm (C6 dermatome), mid-axillary line (T4 dermatome, 5–10 cm from incision, affected side) and iliac crest (L1 dermatome). The pressure pain tolerance thresholds were measured bilaterally on the index finger (C6 dermatome), iliac crest (L1 dermatome) and sternum in the midline (T4 dermatome). To avoid mass significance and as a measure of central sensitization the sum of all the thresholds (SOT) across dermatomes was calculated [14] for the electric thresholds and for the pressure thresholds. Postoperative changes in SOTs were expressed as percentage changes compared to preoperative baseline.

### Conditioned pain modulation (CPM) paradigm

The condition pain modulation paradigm tests the ability to generate descending inhibitory modulation [31]. An electric pain threshold (test stimulus) was determined before and after a cold pressor task (conditioning stimulus), and the CPM effect was determined as the relative change (%) in electric pain threshold. For the cold pressor task the dominant hand was immersed in ice-chilled water (1.0°C ±0.3°C) stirred by pump. The patient was told to remove the hand from the water after two minutes of immersion–or sooner if the pain was considered intolerable–and immersion time was noted. Immediately after the cold pressor task, the subjects rated the pain experienced during the test by VAS for quality control purposes. Electric pain thresholds were obtained in the L1 dermatome immediately before and after ice-water immersion.

### Quality of life

At baseline and 1, 3, 6, and 12 months post surgery patients filled out a quality of life questionnaire (Dutch version of the European Organization for Research and Treatment of Cancer Quality of Life Questionnaire-C30). The EORTC QLQ-C30 is internationally validated for evaluating quality of life in daily living and symptoms and side effects related to different treatment modalities [32]. The individual functional, symptom and quality of life (QOL) scales were summated to create general sum scores [33]. We calculated symptom, functioning and QOL sum scores from the EORTC questionnaires.

### Outcome measures

The primary study outcomes are change in electric and pressure SOT after surgery vs. baseline values. Secondary outcomes are VAS pain and EORTC symptom, functional and QOL sum scores.

### Power-analysis

Based on data from previous postoperative quantitative sensory testing studies by our group [28, 34] we can expect electric pain tolerance thresholds in thoracic dermatomes five days after surgery to be 8.1 mA (SD = 4.5 mA). Sample size calculation based on these data for Type 1 error (alpha) of 0.05 and power (beta) of 80% predicts ability to detect a clinically relevant change in pain tolerance thresholds of one third with a sample size per group of 45 patients. Assuming a drop-out rate of 20–25%, a sample size of n = 55 per group should suffice to detect clinically relevant reductions of one-third in the pain tolerance threshold (vs. the other group) at 5 days postoperatively.

### Data and statistical analysis

Data were analyzed with Statistica (version 12.0, Statsoft, Tulsa, OK, USA), p<0.05 was considered significant. Results are expressed as mean ± 95% confidence interval. Postoperative sums of thresholds were expressed as percentage change compared to preoperative baseline. Chi-squared tests and t-tests were used to assess differences between the treatment groups regarding axillary lymph node dissection, type of surgery, duration of surgery, surgical complications, size of specimen removed, baseline electric SOT, baseline pressure SOT, baseline CPM, baseline QOL-score, baseline functioning score and baseline symptom score.

Our main analysis was aimed at testing our primary hypothesis that perioperative COX-2 inhibition would result in less hyperalgesia as a sign of central sensitization following surgery compared to a placebo-supplemented group. We performed mixed model analyses on change in electric and pressure SOT, and on secondary outcomes VAS scores and EORTC sum scores with fixed factors medication (COX-2 inhibition vs. placebo) and time, and subjects were included as random factor. Preoperative CPM is reported in the literature as a predictor for persistent postsurgical pain development and was included as covariate [35]. We performed a (modified) intention to treat analysis, which included all patients that received at least one dose of study drug (COX-2 inhibition or placebo) [36–39]. Post-hoc tests with Bonferroni correction were used to identify significant differences between medication groups or time levels when a main or interaction effect for the factors was found. A two-sided p-value <0.05 was considered significant for all tests.

To test our secondary hypothesis that patients developing persistent pain 12 months postoperatively would exhibit more hyperalgesia following surgery than patients not complaining of persisting pain two groups were formed post hoc: patients with persistent pain 12 months postoperatively (answering “yes” to the question:” do you have persistent pain due to your surgery”, plus reporting pain at rest or on movement of >30 mm on VAS) or those without persistent pain. The chosen cutoff score >30 mm VAS is widely used in the pain literature [40, 41], corresponds to moderate or severe pain [42], and a 30 mm VAS difference is a relevant treatment difference [43, 44]. We performed additional mixed model analyses on SOTs, pain scores and QOL scores to assess differences between these two groups. Post-hoc tests with Bonferroni correction were used to identify significant differences between patients with and without persistent pain or time levels when a main or interaction effect for the factors was found.

## Results

**Fig 1:**
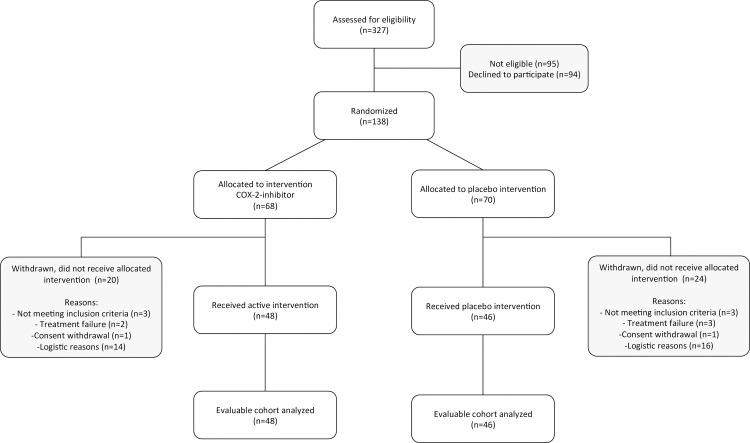
Study enrollment and randomization.

From October 2006 to December 2010 a total of 327 patients were screened for eligibility and 138 patients were randomized (Fig 1). There was relatively high exclusion rate due to treatment failure (unsuccessful paravertebral block) in 5 patients and failure of the hospital pharmacy to deliver study drugs to the operating room on time in 30 patients. 6 patients were excluded because they were found not to be suffering from malignant disease after pathological examination. 94 patients were analyzed in the modified intention to treat analyses. When we compared demographics of excluded patients for mastectomy rate (32 vs. 28%, chi-squared test p = 0.59) and age (56 ± 14 vs. 53 ± 10, unpaired t-test p = 0.27) we found them to be comparable to the analyzed groups.

There were no differences in baseline and demographic data between the placebo and COX-2 inhibition group (Table 1). Surgical complications occurred in 5 patients in the placebo group, consisting of hematomas that had to be drained in 3 patients, an abscess that had to be drained, and a nipple granuloma that had to be removed operatively. One patient in the COX-2 inhibition group developed an infected seroma that had to be drained. Because immediate reconstruction was an exclusion criterion, there was only one patient (in the COX-2 inhibition group) that underwent reconstruction by insertion of a tissue expander and subpectoral prosthesis implantation (495 cc, Mentor^**©**^, Minneapolis, MN, USA) in the year following the initial breast cancer surgery. There were no harmful or unintended effects associated with COX-2 inhibition.

**Table 1:** Demographic data of patients receiving COX-2 inhibition and placebo medication.

	*COX-2 inhibition (n = 48)*	*Placebo (n = 46)*	*P-value*
Age in years	51 ± 9	55 ± 11	0.09
Body Mass Index in kg/m^2^	26 ± 5	25 ± 4	0.46
All mastectomies in %	25	30	0.56
Modified radical mastectomy in %	15	9	0.57
Axillary lymph node dissection in %	35	39	0.71
Duration of surgery in minutes	48 ± 25	44 ± 20	0.39
Size of specimen removed in cm^3^	435 ± 747	437 ± 695	0.99
Surgical complications in %	2	11	0.08
Chemotherapy in %	35	43	0.42
Radiotherapy in %	67	50	0.10
Electric SOT in mA	59 ± 29	58 ± 20	0.86
Pressure SOT in kPa	3167 ± 1397	2797 ± 1102	0.16
CPM in %	40 ± 43	29 ± 35	0.19
Functioning score	82 ± 14	86 ± 12	0.14
Symptom score	11 ± 12	9 ± 7	0.34
QOL score	70 ± 23	76 ± 18	0.23

Data are mean ± sd, continuous data were compared using unpaired t-tests, binomial data using chi-squared tests. SOT, sum of thresholds, CPM, conditioned pain modulation, QOL, quality of life.

### Primary hypothesis: hyperalgesia and COX-2 inhibition

#### Electric and pressure SOT

**Fig 2:**
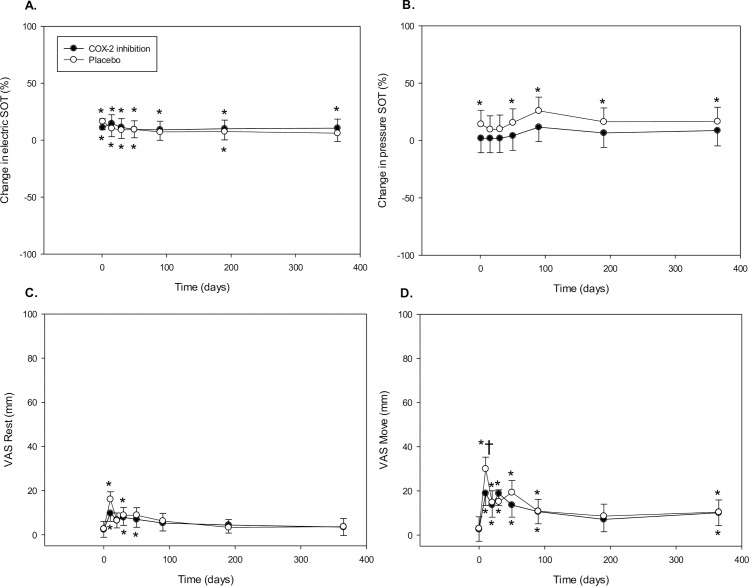
Effect of medication on electric and pressure SOT and VAS scores. Panels A + B show mean change ± 95% CI of SOT versus baseline, panels C + D show the mean ± 95 CI of VAS scores at the different time points. *Different vs. baseline (Bonferroni adjusted p<0.05); †Different vs. COX-2 inhibition (Bonferroni adjusted p<0.05). SOT, sum of thresholds, VAS, Visual Analogue Score.

Perioperative treatment with COX-2 inhibition was not associated with postoperative differences in tolerance to electric or pressure stimulation (Fig 2 and Table 2).

**Table 2:** Results of the main and secondary analyses.

	Main Analysis			Secondary Analysis		
*Factor*	*Medication*	*Time x Medication*	*CPM*	*Persistent pain*	*Time x Persistent pain*	*CPM*
Effect on electric SOT (P-value, effect size)	0.74, 4.42	0.52, N/A	**0.04, 0.11**	0.88, -9.56	0.37, N/A	**0.03, 0.14**
*Pairwise comparisons (P-value*, *effect-size)*						
Day 1	N/A			N/A		
Day 5	N/A			N/A		
Day 15	N/A			N/A		
Month 1	N/A			N/A		
Month 3	N/A			N/A		
Month 6	N/A			N/A		
Month 12	N/A			N/A		
Effect on pressure SOT (P-value, effect size)	0.12, -7.93	0.99, N/A	0.06, 0.16	**<0.01, -30.51**	0.36, N/A	0.07, 0.17
*Pairwise comparisons (P-value*, *effect-size)*						
Day 1	N/A			0.35, -12.68		
Day 5	N/A			**0.02, -30,96**		
Day 15	N/A			**<0.01, -34.97**		
Month 1	N/A			**<0.01, -47.09**		
Month 3	N/A			**0.02, -30.95**		
Month 6	N/A			**<0.01, -41.47**		
Month 12	N/A			**0.03, -30.51**		
Effect on VAS at rest (P-value, effect size)	0.47, -0.18	0.31, N/A	0.61, -0.01	**<0.01, 18.97**	**<0.01, N/A**	0.14, -0.03
*Pairwise comparisons(P-value*, *effect-size)*						
Baseline	N/A			0.57, 2.00		
Day 1	N/A			**<0.01, 22.10**		
Day 5	N/A			0.05, 6.75		
Day 15	N/A			**0.03, 7.74**		
Month 1	N/A			**0.02, 7.98**		
Month 3	N/A			**<0.01, 12.89**		
Month 6	N/A			**<0.01, 14.75**		
Month 12	N/A			**<0.01, 18.97**		
Effect on VAS on movement (P-value, effect size)	0.44, -0.28	**0.02, N/A**	0.70, 0.01	**<0.01, 47.20**	**<0.01, N/A**	0.73, 0.01
*Pairwise comparisons (P-value*, *effect-size)*						
Baseline	0.92, -0.39			0.29, 5.34		
Day 1	**<0.01, -11.09**			**<0.01, 29.41**		
Day 5	0.75, -1.24			**<0.01, 18.71**		
Day 15	0.35, 3.70			**<0.01, 17.03**		
Month 1	0.12, -6.19			**<0.01, 17.66**		
Month 3	0.98, -0.12			**<0.01, 28.83**		
Month 6	0.71, -1.50			**<0.01, 27.22**		
Month 12	0.95, -0.28			**<0.01, 47.20**		
Effect on function score (P-value, effect size)	0.31, -0.52	0.48, N/A	0.39, 0.03	**<0.01, -12.59**	0.38, N/A	0.34, 0.04
*Pairwise comparisons (P-value*, *effect-size)*						
Baseline	N/A			0.19, -6.52		
Month 1	N/A			0.06, -9.47		
Month 3	N/A			**<0.01, -15.91**		
Month 6	N/A			**<0.01, -13.29**		
Month 12	N/A			**0.02, -12.59**		
Effect on symptom score (P-value, effect size)	0.30, -0.25	0.21, N/A	0.73, -0.01	**<0.01, 16.69**	**<0.01, N/A**	0.41, -0.02
*Pairwise comparisons (P-value*, *effect-size)*						
Baseline	N/A			0.24, 4.08		
Month 1	N/A			**<0.01, 9.56**		
Month 3	N/A			**<0.01, 16.37**		
Month 6	N/A			**<0.01, 13.05**		
Month 12	N/A			**<0.01, 16.69**		
Effect on QOL score (P-value, effect size)	0.28, 0.04	0.57, N/A	0.53, 0.02	**<0.01, -14.14**	0.14, N/A	0.34, 0.03
*Pairwise comparisons (P-value*, *effect-size)*						
Baseline	N/A			0.14, -6.13		
Month 1	N/A			**0.02, -9.56**		
Month 3	N/A			**<0.01, -15.55**		
Month 6	N/A			**<0.01, -12.28**		
Month 12	N/A			**<0.01, -14.14**		

SOT, sum of thresholds, VAS, vusual analogue scale, QOL, quality of life. P-values for post-hoc test were adjusted for multiple testing using Bonferroni correction.

#### VAS scores

COX-2 inhibition did not affect VAS scores at rest but influenced VAS scores on movement (Time x Medication: p = 0.02)–Fig 2 and Table 2. Post-hoc testing revealed that COX-2 inhibition led to lower postoperative VAS score on movement only on postoperative day 5.

#### Preoperative CPM

Covariate preoperative CPM significantly affected electric SOT (p = 0.04), and showed a trend towards significance on pressure SOT (p = 0.06)–Table 2. Impaired preoperative CPM was related to more negative postoperative change in sensitivity. Of note, preoperative CPM did not influence postoperative VAS scores at rest or on movement.

#### EORTC sum scores

**Fig 3:**
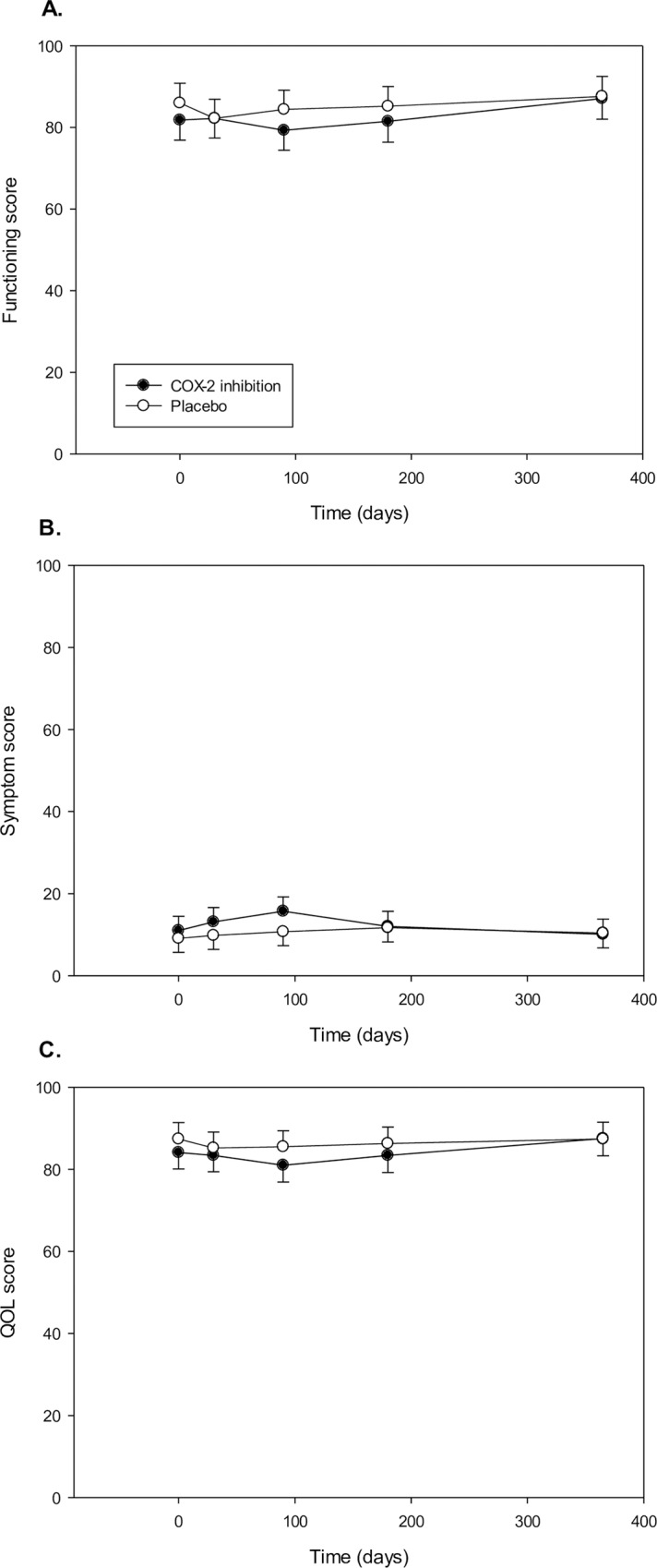
Effects of medication on EORTC function, symptom and QOL sum scores. Panels A-C show the mean ± 95% CI at the different time points. No difference between treatment groups at any time (all p>0.05). QOL, quality of life.

EORTC functioning, symptom and QOL score were comparable between treatment groups (Fig 3 and Table 2).

### Secondary hypothesis: persistent pain and hyperalgesia

#### Persistent postsurgical pain

Twelve months postoperatively 11 patients (13%) complained of persistent pain with VAS>30 mm. Characteristics of patients that eventually would develop persistent pain and patients free of pain are displayed in Table 3. Patients that would eventually develop persistent pain had higher baseline pressure SOT and electric SOT.

**Table 3:** Baseline characteristics of patients that eventually developed persistent and patients without pain.

*With/Without persistent postsurgical pain*	*With persistent pain (n = 11)*	*Without persistent pain (n = 83)*	*P-value*
Age in years	53 ± 7	54 ± 10	0.79
Body mass index in kg/m^2^	27 ± 7	26 ± 4	0.37
All mastectomies in %	36	27	0.53
Modified radical mastectomy in %	27	8	0.17
Axillary lymph node dissection in %	54	36	0.23
Duration of surgery in minutes	46 ± 22	53 ± 27	0.34
Size of specimen removed in cm^3^	508 ± 872	459 ± 741	0.84
Surgical complications in %	9	6	0.70
Chemotherapy in %	45	39	0.66
Radiotherapy in %	64	60	0.82
Electric SOT in mA	81 ± 37	56 ± 22	**<0.01**
Pressure SOT in kPa	4114 ± 1416	2821 ± 1224	**<0.01**
CPM in %	28 ± 56	33 ± 33	0.65
Functioning score	78 ± 16	85 ± 13	0.12
Symptom score	14 ± 12	9 ± 9	0.20
QOL score	80 ± 13	87± 11	0.09

Data are mean ± sd, continuous data were compared using unpaired t-tests, binomial data using chi-squared tests. SOT, sum of thresholds, QOL, quality of life, CPM, conditioned pain modulation.

#### Electric and pressure SOT

**Fig 4:**
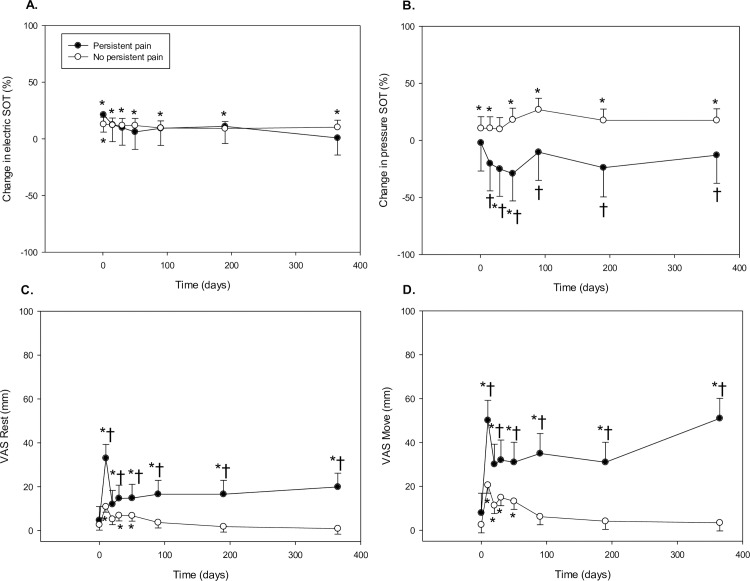
Electric and pressure SOT and VAS scores in persistent pain patients versus women without pain. Panels A + B show mean change ± 95% CI of SOT versus baseline, panels C + D show the mean ± 95% CI of VAS scores at the different time points. *Different vs. baseline (Bonferroni adjusted p<0.05); † Different vs. No persistent pain (Bonferroni adjusted p<0.05). SOT, sum of thresholds, VAS, Visual Analogue Score.

Patients in the persistent postsurgical pain group did not exhibit postoperative hyperalgesia to electric stimulation, but were significantly more hyperalgesic postoperatively to pressure stimulation (Persistent pain: p<0.01)–Fig 4 and Table 2. Post-hoc analysis revealed that persistent pain patients were hyperalgesic to pressure stimulation versus patients not developing pain on day 5 and throughout the rest of the postoperative year.

#### VAS scores

Persistent pain patients had significantly higher postoperative pain VAS scores at rest (Persistent pain: p<0.01, Time x Persistent pain: p<0.01) and on movement (Persistent pain: p = <0.01, Time x Persistent pain: p<0.01)–Fig 4 and Table 2. These differences existed at all early and late postoperative timepoints, except for VAS at rest on day 5. Paravertebral blockade provided excellent postoperative pain relief in the patients not developing persistent postsurgical pain.

#### Preoperative CPM

Covariate preoperative CPM significantly affected electric SOT (p = 0.03) and showed a trend towards significance on pressure SOT (p = 0.07)–Table 2. Impaired preoperative CPM was related to more negative postoperative change in sensitivity. Preoperative CPM did not influence postoperative VAS scores at rest or on movement.

#### EORTC sum scores

**Fig 5:**
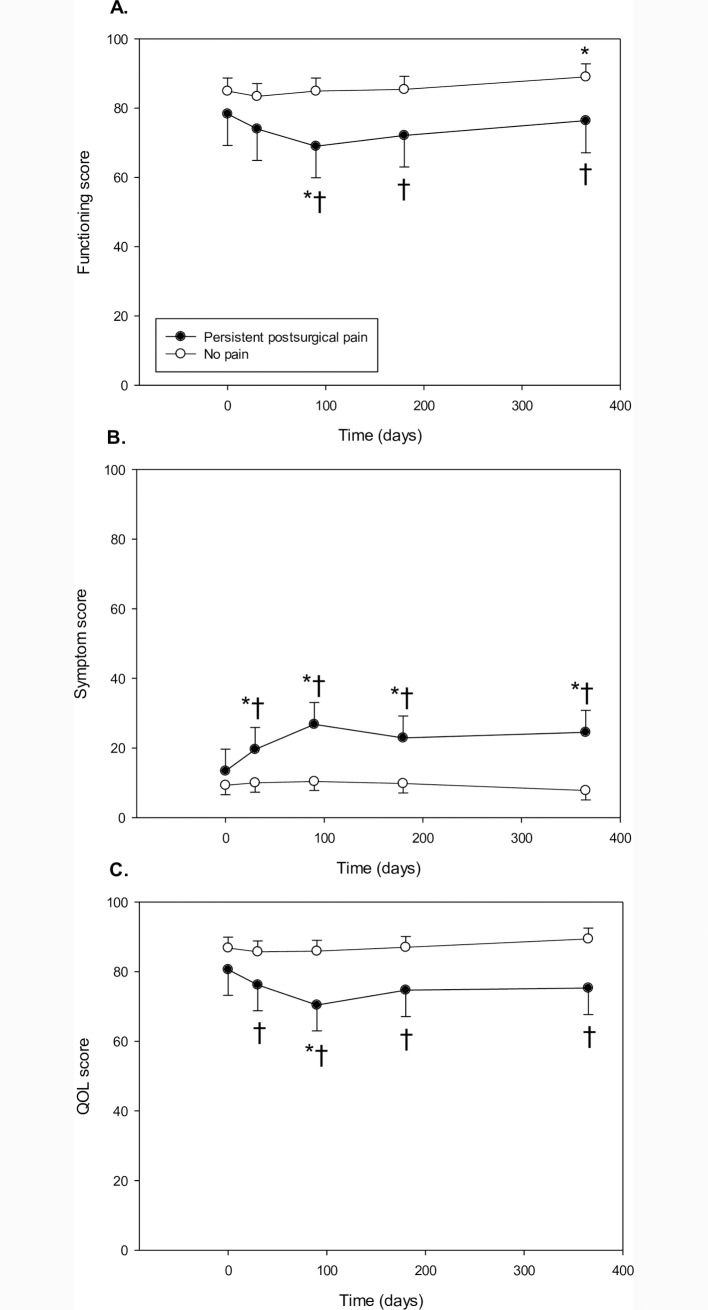
EORTC function, symptom and QOL sum scores in persistent pain patients versus patients with no pain. Panels A-C show the mean ± 95 CI at the different time points. *Different vs. baseline (Bonferroni adjusted p<0.05); † Different vs. No persistent pain (Bonferroni adjusted p<0.05). QOL, quality of life.

Patients in the persistent postsurgical pain group reported lower functioning score (Persistent pain: p<0.01) and total QOL (Persistent pain: p<0.01), and higher symptom score (Persistent pain: p<0.01, Time x Persistent pain: p<0.01)–Fig 5 and Table 2. Lower functioning score was present at 3, 6 and 12 months postoperatively for persistent pain patients. Persistent pain was associated with higher symptom score and lower total QOL score at 1, 3, 6 and 12 months postoperatively.

## Discussion

We assessed the value of inhibiting the inflammatory component of sensitization added to blockade of neuronal nociceptive input (paravertebral blockade) on central sensitization (expressed as widespread hyperalgesia) and persistent pain after surgery in women undergoing surgery for breast malignancy. Adding perioperative COX-2 inhibition to maximal anti-nociceptive therapy had no impact on change in electric or pressure pain tolerance thresholds as a measure of central sensitization after breast cancer surgery. COX-2 inhibition did lead to lower pain scores on movement at postoperative day 5, but had no effect on later time points and did not affect quality of life scores. Thus, our primary hypothesis was rejected.

We found that patients developing persistent postsurgical pain were significantly more hyperalgesic to pressure both early after surgery (5 days) and throughout the rest of the year (15 days to 12 months). Thus, our secondary hypothesis was confirmed. There was no difference in sensitivity to electric quantitative sensory testing. Patients with persisting pain 12 months postoperatively had more pain in the acute postoperative period (1, 5 and 15 days) and the rest of the year (1, 3 and 6 months). Total QOL and functioning scores were lower in persistent pain patients and symptom score was higher vs. patients not developing persisting pain.

Our study describes the effects of perioperative COX-2 inhibition on postoperative sensitization of pain processing in a long-term prospective and longitudinal trial. Regarding persistent pain (but not hyperalgesia) after breast surgery some data are available regarding perioperative COX-2 inhibition. Romundstad et al. [45] found no difference versus placebo of a single peri-operative dose of 40 mg parecoxib on persistent pain one year after surgery in patients undergoing augmentation mammaplasty. Another trial [46] reported no impact on pain six months postoperatively of ibuprofen 400 mg before mastectomy plus four additional doses afterwards.

Surgical tissue damage is associated with prostanoid production [47]. This release, involving COX-2 induction, occurs peripherally and in the central nervous system [24]. Peripheral release of prostanoids (PGE-2, PGI-2) sensitizes peripheral nociceptors. Centrally synthesized PGE-2, by increased COX-2-expression, leads directly to central sensitization of the pain system [24, 48, 49]. COX-2 inhibitors interfere with both the peripheral and the central prostaglandin production [27]. Therefore, perioperative inhibition of COX-2 was expected to ameliorate central sensitization and to increase pain thresholds, by both inhibiting peripheral nociceptive input and by inhibiting direct central sensitization under influence of prostaglandins. We did not observe this expected difference, suggesting that perioperative COX-2 induction and inflammation subsequent to tissue damage may be of little importance in inducing postoperative central sensitization. Interestingly, a small recent trial with parecoxib failed to induce a difference in pressure pain tolerance thresholds in CPRS patients outside the surgical context [50].

The generalized pressure hyperalgesia detected in this study suggests that persistent central sensitization is an important process in persisting pain development after surgery. Other quantitative sensory testing studies have assessed pain processing in women with persistent pain at single time-points after breast cancer surgery. These studies confirm the widespread mechanical hyperalgesia we observed [51, 52]. Others have demonstrated enhanced sensitivity to electrical and thermal stimulation [53, 54], further supporting the presence of central sensitization in persistent pain after breast cancer treatment. Recently, Andersen et al. found a relationship between sensory disturbances and pain one week after surgery for breast cancer [55]. Hyperalgesia has also been reported 5 days after back surgery [56] and early postoperative hyperalgesia has been linked to persistent pain development in smaller studies after abdominal surgery [57, 58]. Hyperalgesia in the postoperative period is likely to be expressed as increased pain experience, which we found for both VAS at rest and on movement. A significant relationship between early postoperative pain and persistent pain has previously been reported for breast cancer surgery [13] and other interventions including cholecystectomy [8, 9], groin hernia repair [10] and thoracic surgery [12].

We observed a relatively low incidence of persistent postsurgical pain following surgery (13%) compared to other less recent studies (25 to 60%)[59]. Maximal peri-operative blockade of neuronal nociceptive input (paravertebral blockade), but also identification and attention to sparing intercostobrachial and other nerves during lymph node dissection in the present study [22], may explain the low incidence of persistent pain.

### Implications

Our results indicate that the role of COX-2 and inflammation in the genesis of postoperative hyperalgesia may be less important than that of neuronally mediated nociceptive input. Adding perioperative COX-2 inhibition to maximal neuronal anti-nociceptive therapy (paravertebral blockade) appears of limited clinical value in preventing postoperative hyperalgesia or persistent pain.

Despite all patients receiving maximal neuronal anti-nociceptive therapy in the form of paravertebral blockade, 13% of patients still developed persistent pain postoperatively. These patients showed widespread hyperalgesia to pressure in the acute postoperative period and the rest of the year and would seem to be relatively resistant to current therapeutic interventions. Future studies should further explore causes of developing early and persistent postoperative hyperalgesia, possibly an important process during persistent pain development.

Clinically, the fact that persistent pain patients showed more widespread hyperalgesia to pressure in the acute postoperative period means that peri-operative monitoring using quantitative sensory testing should be able to identify patients at risk of developing persistent postsurgical pain, possibly allowing for targeted antihyperalgesic treatment.

There is increasing interest in the potential for perioperative quantitative sensory testing to predict persistent postsurgical pain. A relationship between peri-operative quantitative sensory testing measures and persistent pain has thus far been shown in only a limited number of studies. These studies demonstrated an association between persistent postsurgical pain and preoperative measures of widespread pain sensitization, such as pressure pain thresholds [60–63]. However most of these studies were conducted in the context of orthopedic joint surgery and represent a very different patient population. In the orthopedic population patients have often suffered from ongoing pain and nociceptive input for a prolonged time preoperatively, which may have lead to sensitized central pain processing even before surgery. Conversely, patients undergoing surgery for breast cancer are highly unlikely to have suffered from significant pain preoperatively and are thus unlikely to express centrally sensitized pain processing preoperatively. Future studies should clarify which quantitative sensory measurement at which time point can best predict persistent pain in the breast cancer population.

Prediction and possible interventions targeting persistent pain after breast cancer surgery are especially relevant given the poorer function scores and QOL we found with persistent pain after breast cancer surgery. Furthermore, persistent pain after breast cancer surgery is increasing in prevalence due to increased survival after breast cancer [59].

### Methods and limitations

We chose quantitative sensory testing measures as the main outcome measures because we intended to conduct a study investigating relations between COX-2 inhibition, hyperalgesia development and persistent pain after surgery. Pressure quantitative sensory testing detects hyperalgesia of deep tissues such as muscle as a manifestation of central sensitization [30], and is considered a clinically robust and reliable measurement [64]. Electric quantitative sensory testing stimulates peripheral cutaneous nerve endings bypassing peripheral nociceptors in skin and is sensitive to local and descending modulation [29]. These characteristics may explain why we found differences in pressure tolerance thresholds–but not in electric tolerance thresholds–in persistent pain patients.

We measured electric and pressure pain tolerance thresholds at multiple topographic sites. Others have advocated extensive multimodal quantitative sensory testing protocols [65]. These protocols permit quantification of several different aspects of hyperalgesia, without, however, achieving testing altered sensitivity at multiple sites, and are time-consuming. We chose our battery of tests for its suitability for implementation into clinical practice. This testing protocol generally lasts about 30 minutes and is well-accepted by patients with good reproducibility (within 20%)[66].

Two limitations pertain to this study. First, we were unable to include some patients in the modified intention to treat analysis due to treatment failure, and had to exclude some patients due to an incorrect initial diagnosis. However, we achieved the group size we calculated beforehand to deliver sufficient power to detect a 30% difference from baseline and the analyzed treatment groups were comparable for baseline characteristics. A second limitation was the small number of patients developing pain 12 months postoperatively. A larger study population and thus a larger group of pain patients might have provided more insight into the deferential characteristics of persistent pain patients vs. patients not suffering from pain, even though we were able to detect several significant differences between the groups that we analyzed.

## Conclusions

In conclusion, we found that adding perioperative COX-2 inhibition to current maximal anti-nociceptive therapy (paravertebral blockade) has no significant impact on central sensitization, persistent pain and QOL in the year following breast cancer surgery. Patients that developed persistent pain after breast cancer surgery were significantly more hyperalgesic, had higher pain scores and lower QOL throughout the year following surgery. Sensitization early after surgery may play a role in the genesis of persistent pain after breast cancer surgery and perioperative monitoring using quantitative sensory testing may be able to identify patients at risk of developing persistent pain.

## Supporting Information

S1 Checklist (DOC)

S1 Protocol (DOC)
