# Supplementary material for: Hyperalgesia and Persistent Pain after Breast Cancer Surgery: A Prospective Randomized Controlled Trial with Perioperative COX-2 Inhibition
Source: PLoS One. 2016 Dec 9;11(12):e0166601. doi: 10.1371/journal.pone.0166601 (PMC5147830; doi:10.1371/journal.pone.0166601)
Supplement: S1 Protocol — (DOC) [file pone.0166601.s002.doc]

# **Relative Contributions of Humoral and Nerve Nociceptive Inputs to Postoperative Central Sensitisation and Pain:**

# Interaction of Extended Perioperative COX-2 Inhibition and Local Anaesthetic Blockade for Breast Malignancy Surgery

## **Principal Investigator**

## Oliver H.G. Wilder-Smith ^1^

## **Investigators**

## Monique Steegers ^1^

## Carel Slegers [[1]](#footnote-2)2

## **Researchers**

## Daphne Snik

## Sanna Rijpma

## Rianne van Boekel

## **Affiliations**

## ^1^ Pain Centre, Department of Anaesthesiology,

## UMC St. Radboud, POB 9101

## NL-6500 HB Nijmegen, The Netherlands

## ^2^Department of Anaesthesiology and Surgery

*Ziekenhuis Bernhoven*

*NL-5342 BT Oss, The Netherlands*

### Table of Contents

### 1. Summary

### 2. Rationale

### 3. Background Information

### 4. Trial Objectives

### *4.1 Primary Objective*

### *4.2 Primary Endpoint*

### *4.3 Secondary Objectives*

### *4.4 Secondary Endpoints*

### 5. Trial Design

### *5.1 Study Design*

### *5.2 Study Population*

### *5.3 Recruitment*

### *5.4 Exclusion Criteria*

### *5.5 Treatment*

### *5.6 Measures and Endpoints*

### *5.7 Times of Measures*

### *5.8 Blinding*

### *5.9 Power Analysis*

### *5.10 Analysis and Statistics*

### 6. Withdrawal

### *6.1 Individual Stop Criteria*

### 7. Ethics

### *7.1 Ethical Conduct of Trial*

### *7.2 Subject Information and Consent*

### *7.3 Study Drugs*

### 8. References

### 9. Appendices

### *9.1 Subject Information and Consent Form*

### *9.2 Quantitative Sensory Testing Protocol*

### 1. Summary

Breast surgery for cancer is associated with both tissue and nerve damage, and is frequently followed not only by acute, but also by chronic pain, causing significant medical and social morbidity. The nociception of surgery is now recognised to result in postoperative hyperalgesia via changes in central nervous system processing (central sensitisation). Such neuroplasticity is mediated both via humoral (tissue damage leading to inflammation) and neuronal (nerve damage leading to neuropathy) nociceptive inputs. Postoperative hyperalgesia will lead to increased pain, which – together with nerve damage – has recently been linked to subsequent pain chronification in a number of studies. Based on animal studies, it is now accepted that a major mechanism for such pain chronification is the persistence of (abnormal) central sensitisation.

The aim of this study is to investigate the interaction between extended perioperative COX-2 inhibition (starting before surgery and continuing 5 days into the postoperative period) and extended locoregional block (paravertebral block) using local anaesthetics on central sensitisation after breast surgery. This should enable us to gain insight into the relative contributions of humoral (inflammatory) and neuronal (neuropathic) nociceptive inputs to acute postoperative hyperalgesia, persistence of central sensitisation and pain chronification after breast surgery. The defined primary study endpoint is the persistence and extent of central sensitisation one month after breast surgery as measured by quantitative sensory testing. The study will further investigate effects on clinical pain and other outcome measures, and determine their relationship to measures of central sensitisation.

### 2. Rationale for study

### Pain is common after breast surgery for malignancy. Both acute and chronic pain significantly affect quality of life and are thus undesirable of themselves. Moreover, pain can potentially affect other long-term outcomes of cancer surgery in the form of increased recurrence coupled with decreased survival via concomitant effects on the immune system (Liebeskind 1991, Bar-Yosef et al 2001).

A key insight from fundamental pain research over the last decades has been the finding that nociceptive input alters subsequent sensory processing by the nervous system (Coderre et al 1993). Initially, this neuroplasticity is excitatory (“central sensitisation” for the central nervous system), thus resulting in increased sensitivity to pain (“hyperalgesia”) (Woolf & Salter 2000). Hyperalgesia after surgery is the result of two separate mechanisms, namely damage to tissues and to nerves, with the former acting more via humoral (biochemical products of tissue inflammation), and the latter more via neuronal (via nerve damage and stimulation) mechanisms (Coderre et al 1991, Woolf & Salter 2000, Wieseler-Frank et al. 2004). The relative contribution of the two mechanisms has not been investigated to date.

Tissue damage due to surgery is associated with the production of prostanoids (Samad et al. 2002). This release, involving induction of COX-2, occurs not only peripherally but also in the central nervous system (Samad et al. 2001). Peripherally, the production of prostanoids sensitises peripheral nociceptors. Centrally, sensitisation and the accompanying sickness syndrome (fever, lethargy, diffuse pain, anorexia, etc) are induced by interleukin-1 beta causing the upregulation of COX-2 resulting in elevated prostaglandin E2 levels in the cerebrospinal fluid. This central response to inflammation can be inhibited by preventing central prostanoid production via block of interleukin-converting enzyme or COX-2, but not COX-1 (Samad et al. 2001, Svensson & Yaksh 2002). Furthermore, a synergistic action between central and peripheral COX-2 inhibition has been suggested and demonstrated in this context (Samad et al. 2001). The effectiveness of extended perioperative COX-2 blockade in affecting postoperative pain and functional outcomes has recently been demonstrated (Buvanendran et al 2003)

The fact that neuronally carried nociceptive input from surgery is associated with central sensitisation is well-known and described, as is the fact that local anaesthetic blockade is an effective means of blocking such input (Coderre et al 1993).

Acute hyperalgesia after surgery is clearly undesirable of itself, as it will increase the amount of pain felt for a given nociceptive input. More pain means more stress, thus increasing the metabolic demands made on the body, resulting in increases in a variety of complications (Rodgers et al 2000). Furthermore, increased pain will also increase the impact of the pain on the immune system, a clearly unwanted consequence (Bar-Yosef et al 2001). Finally, more pain usually means more demand for analgesics, which have their own list of side-effects, many of them again unwelcome (e.g. opioid side-effects).

However, the effects of postoperative hyperalgesia do not remain limited to the acute period. As already mentioned, chronic pain syndromes are now recognised to be much more common than initially thought after surgery (Perkins & Kehlet 2000, Macrae 2001). It is also generally accepted today that the mechanism of pain chronification involves persistent and abnormal excitatory neuroplasticity after nociception (Coderre et al 1993, Woolf & Salter 2000). The details of this process remain incompletely understood and studied, particularly in the postsurgical context. Two main somatic risk factors for pain chronification after surgery have been identified in the literature from clinical studies to date: nerve damage and prolonged/abnormal pain in the early postoperative period (Perkins & Kehlet 2000, Macrae 2001). Nerve damage of itself can cause early postoperative hyperalgesia, and persistent and abnormal hyperalgesia in the early postoperative period – the result of nerve as well as tissue damage – is likely to be expressed as prolonged/abnormal pain in this period. **In the context of the identification of persistent excitatory neuroplasticity as a basal mechanism of pain chronification, this makes it likely that the prevention of early postoperative hyperalgesia (due to sensitisation resulting from tissue and nerve damage) – and of its persistence – will play and important role in the subsequent prevention of pain chronification.**

### For improved therapeutic management of the acute and chronic pain accompanying breast surgery it will be useful to better understand the relative contributions of tissue and nerve damage to the causation of acute and chronic pain. Breast surgery can involve both damage to soft tissues (the breast, inflammation, humoral input) as well as nervous structures (axillary sampling, neuropathy, neuronal imput). Thus this type of surgery permits us to study the question of differential involvement of inflammatory and neuropathic processes in acute and chronic pain outcomes by 1) observing the effects of breast surgery with or without axillary surgery, and 2) by observing the effects of drugs specifically modulating inflammatory effects (COX-2 inhibitors) in addition to the effect of techniques specifically modulating neuropathic effects (local anaesthetics).

The aim of this study is to investigate the interaction between extended perioperative COX-2 inhibition (starting before surgery and continuing 5 days into the postoperative period) and extended locoregional block (paravertebral block) using local anaesthetics on excitatory neuroplasticity after breast surgery. This should enable us to gain insight into the relative contributions of humoral (inflammatory) and neuronal (neuropathic) inputs to acute postoperative hyperalgesia, persistence of central sensitisation and pain chronification after breast surgery. The defined primary study endpoint is the persistence and extent of central sensitisation one month after breast surgery as measured by quantitative sensory testing. The study will further investigate effects on clinical pain and other outcome measures, and determine their relationship to measures of central sensitisation.

### 3. Background Information

One year after mastectomy, a recent survey quoted 43-56% of patients as suffering from chronic pain, with 26-45% reporting neuropathic problems in the arm and 26-66% describing phantom breast pain (Tasmuth et al. 1999). High incidences of pain complications are also described in other publications (e.g. Carpenter et al 1998, Wallace et al 1996, Gottrup et al 2000). Pain complications after mastectomy are a significant source of morbidity and are significantly related to reductions in the quality of life (Tasmuth et al. 1999). More severe acute pain after various types of surgery has been linked to the development of late, chronic pain complications (Tasmuth et al. 1999, Tasmuth et al. 1997, Katz et al. 1996, Kalso et al. 1992, Dworkin et al. 1992). Pain in the cancer context has also been linked to impaired immune function and thus poorer survival (Bar-Yosef S et al 2001, Liebeskind 1991).

Quantitative sensory testing (QST) permits us to quantify nervous system input-response relationships in humans, allowing detection and quantification of neuroplasticity after nociception. QST thus provides insight into nociceptive mechanisms, with the potential to provide the diagnostics necessary for mechanism-based approaches to perioperative nociception and pain management. In several studies, we have demonstrated that QST based upon electric transcutaneous stimulation is feasible for the demonstration of neuroplasticity in the perioperative clinical context (Wilder-Smith et al 1996, 1998, 2002, 2003).

Parecoxib is an injectable member of the new generation of highly selective COX-2 inhibitors. Parecoxib 20-40mg i.v. has been demonstrated to possess an analgesic efficacy at least equal to 4 mg morphine or 30 mg ketorolac i.v. in the perioperative context (Rasmussen et al 2002, Barton et al 2002). Preliminary animal data suggest that parecoxib crosses the blood brain barrier (data on file, Pfizer Inc., USA). Celecoxib, also a highly selective COX-2 inhibitor, has been shown to be safe and efficacious for perioperative analgesic use in doses of, typically, 2 X 200mg p.o. per day (Straube et al 2005, Chen et al 2004)

Both parecoxib and celecoxib are licensed as analgesics in the Netherlands. Parecoxib is approved for short-term use (e.g. perioperatively) in the Netherland at doses up to 40 mg twice daily. Celecoxib is approved for long-term oral use in the Netherlands, and the doses envisaged in the present study (2 X 200 mg/day) fall within the approved range.

Paravertebral block is a well-established technique of achieving unilateral blockade of the upper trunk of the body (Karmakar 2001). It has been shown to be effective in reducing pain after breast surgery (Buckenmaier et al 2003, Kairaluoma et al 2004). Ropivacaine is commonly used and described in the literature for paravertebral blockade (Karmakar et al 2005), and is registered for long-lasting local anaesthetic block in the Netherlands.

### 4. Trial Objectives

### *4.1. Primary objectives*

- To study the effect of the interaction of cyclooxygenase-2 (COX-2) inhibition (started preoperatively and continued 5 days into the postoperative period) and extended local anaesthetic blockade via paravertebral block on postoperative central sensitisation after breast surgery for malignancy

*4.2. Primary endpoint*

### Neuroplasticity, e.g. central sensitisation as demonstrated by quantitative sensory testing, at one month after surgery

#### *4.3. Secondary objectives*

- To demonstrate that COX-2 inhibition also has favourable effects on postoperative *clinical* outcomes including acute and chronic pain, sickness syndrome, side-effects of analgesia and surgical complications
- To demonstrate that the use of COX-2 inhibition in this context is safe and associated with an acceptable side-effect profile

*4.4. Secondary endpoints*

- Neuroplasticity at other time points after surgery
- Acute and chronic clinical pain measures (pain scores, analgesic consumption, pain questionnaires)
- Other pain outcome measures (incidence of phantom pain, pain maps, analgesia complications)
- Surgical outcome measures (complications)
- Patient satisfaction and well-being (including nausea and vomiting, opioids symptom distress score SDS)

#### 5. Trial Design

*5.1. Study design*

Prospective, randomised and double-blinded. Patients will be prospectively randomised into active (parecoxib and celecoxib) or placebo treatment groups.

*5.2. Study population*

110 patients (n=55 per group) due to undergo elective first surgery for malignancy of the breast at UMC St. Radboud or Bernhoven Ziekenhuis in Oss.

*5.3. Recruitment*

The patients will be recruited during their initial surgical outpatient visit (mammacare). At this time preliminary verbal consent will be obtained and patients will receive detailed written patient information on the trial for further study and reflection. This information also warns the patient that they will be contacted later by telephone regarding possible participation in this trial. About one week later, patients will be contacted by telephone and asked if they wish to participate in the trial. At this time, patients will also be given more details on the study, and will have the opportunity of asking questions. If the patient agrees, he will be instructed as to the further organisation of the study.

At the subsequent anaesthesia outpatient visit (for premedication) formal, written consent will be obtained by the anaesthesiologist and the research nurse, who will also answer questions and perform the demonstration and training necessary for quantitative sensory testing. Inclusion criteria include first elective breast surgery, as well as ability to comply with testing procedures and to give informed consent. Recruitment will be organised in such a fashion as to ensure equal distribution of large and small breast surgery (i.e. mastectomy vs. lumpectomy) and nerve-damaging or non-nerve-damaging surgery (i.e. presence or absence of axilllary surgery) between the two treatment groups.

#### *5.4. Exclusion criteria*

Previous breast surgery, any type of chronic pain syndrome, regular analgesic medication (including opioids, NSAIDs or COX-2 inhibitors) for the 2 weeks preceding surgery, pre-existing central nervous system pathology (e.g. stroke, dementia), conditions predisposing to neuropathy (e.g. diabetes mellitus, alcohol abuse), inability to comply with testing procedures or to give informed consent, presence of contra-indications to COX-2 therapy (e.g. impaired renal or cardiac function (including angina pectoris), untreated hypertension, active or recent gastrointestinal ulceration), contraindications to paravertebral blockade.

*5.5. Treatment intervention*

After informed patient consent, patients will be randomised to either an active or placebo treatment group. On the morning of surgery, the patient will receive oral midazolam premedication (7.5 mg). In the operating theatre, the patients randomised to the active group will receive an i.v. injection of parecoxib 40 mg 30 minutes before the start of surgery. The injection will be repeated 6 hours later. On the evening of the operation day patients will start celecoxib 2 X 200mg/d and continue this scheme until the morning of the fifth postoperative day. The placebo group will receive placebo injections and tablets according to the same regime. As the medication will be blinded, neither observers nor persons involved in patient management nor observers will be aware of which group the patients are in.

#### Anaesthesia

Patients can receive the usual oral benzodiazepine premedication on the evening before (e.g. oxazepam 10 mg p.o.) surgery. On the morning of surgery premedication will consist of midazolam 7.5 mg p.o.. Paravertebral blockade will be performed using the standard technique and 20 ml ropivacaine 0.75%. Before surgery, adequate local anaesthetic blockade will be tested for clinically using pin-prick. Unsuccessful block will lead to exclusion of the patient from the study. General anaesthesia will be via a standard scheme, with anaesthesia being induced using propofol 2-3 mg/kg, fentanyl 3 µg/kg and rocuronium 0.5 mg/kg. Anaesthesia maintenance will be via air/oxygen (40%) and isoflurane. If the procedure lasts longer than 45 minutes from intubation, further analgesic supplementation with fentanyl 1 µg/kg will be permitted at 45 minutes and then at further 45 minute intervals. The last dose of fentanyl should be given no less than 45 minutes before the end of surgery. No further myorelaxant supplementation will be given and no antagonisation will be performed at the end of surgery. The maintenance of a comparable and acceptable depth of anaesthesia using isoflurane will be guided by keeping haemodynamic values to within 20% of preoperative baseline values.

#### Analgesia

In the recovery room, patients will be titrated to VAS≤3 by the recovery room nurse using 3 mg intravenous increments of piritramide as soon as they complain of pain. Standard postoperative analgesia from this time onwards will consists of a fixed paracetamol scheme (4 X 1g paracetamol/day) together with on-demand tramadol (as drops, maximum 300 mg/day) up to day 5 postoperatively. Postoperative nausea and vomiting will be treated by 1) 10 mg metoclopramide i.v., 2) 1.25 mg droperidol i.v. and 3) 4 mg ondansetron i.v. (in that order of escalation).

*5.6. Measures and endpoints*

## Perioperative Measures

1. Time to first request for analgesia in recovery room and total titration dose (piritramide, once only!)
2. VAS (100 mm unanchored visual analogue scale) for somatic and phantom pain intensity (both: at rest, on coughing, on moving arm)
3. Cumulative on-demand tramadol consumption
4. Standard haemodynamic measures, temperature
5. Quantitative sensory testing (QST) by transcutaneous electrical and mechanical stimulation (cf. appendix)
6. Short MPQ (Dutch language version) for somatic and phantom pain
7. Complications, need for drop-out (also an endpoint), patient satisfaction and well-being (including nausea/vomiting, opioid symptom distress score [SDS] and clinically meaningful events score [CME])

#### Additional Documentation

Preoperatively we document smoking habits of the patients as well as demographic variables. Any postoperative complications will be noted. At each data collection point, complications attributable to COX-2 inhibitors will be looked for (allergy, bleeding, gastrointestinal or renal complications). Furthermore, the opioid Symptom Distress Score (SDS) and the Clinically Meaningful Events Score (CME) will be collected at each data collection point.

*5.7. Times of measures*

- At formal recruitment before surgery
- On leaving recovery unit postoperatively (inpatient; not measures 6-7)
- 5, 15 days postoperatively (outpatient)
- 1, 3, 6 and 12 months post-mastectomy (outpatient)
- 12 months post-mastectomy, we will additionally collect details on

1. surgical complications
2. details of adjuvant therapy (chemotherapy, radiotherapy, hormone therapy)
3. wound healing and disease state (recurrence, metastases, progression)

*5.8. Blinding*

The pharmacy of UMC St. Radboud/Ziekenhuis Bernhoven will hold and administer the randomisation scheme for the trial. Parecoxib and celecoxib (active or placebo) will be supplied by the hospital pharmacy.

*5.9. Power analysis*

Based on data from previous postoperative QST studies (Wilder-Smith et al 1996, 1998) we can expect pain tolerance thresholds in thoracic dermatomes five days after surgery to be about 8.1 mA with a standard deviation of about 4.5 mA. Sample size calculation based on these data for a Type 1 Error (alpha) of 0.05 and a Power (beta) of 80% predicts that we should be able to detect a clinically relevant change in paint tolerance thresholds of one third with a sample size per group of 45 patients. Assuming a drop-out rate of 20-25%, the sample size of n=55 per group should thus be large enough to detect a clinically relevant reduction of ca. one-third in the pain tolerance threshold (compared to the other group) at 5 days postoperatively.

*5.10. Analysis and statistics*

The following aspects will be analysed within and between the treatment groups:

- topography of neuroplasticity
- postoperative time course of neuroplasticity
- postoperative time course of clinical pain measures (VAS, MPQ, analgesic consumption) and other clinical outcomes scores (e.g. opioid symptom distress scores)
- presence/absence/severity of phantom pain, pain complications, medical outcomes
- QST vs. pain measures; early QST/pain measures vs. late QST/pain measures vs. medical/pain outcomes
- effects of factor scoring for extent of axillary/breast surgery

Statistical analysis, after testing for normal distribution of data, will be via repeated measures ANCOVA (covariant = initial threshold; factors: scoring for extent of axillary/breast surgery, treatment group, time, measurement site) for change in thresholds, VAS value (somatic/phantom, at rest/on movement) and analgesia consumption. Demographic data will be compared using Student’s t-testing; ordinal data (e.g. scores) will be compared via Kruskal-Wallis ANOVA. Correlations will be assessed via multiple linear regressions, log-transformed if necessary to achieve linearity.

6. Withdrawal

#### *6.1. Individual stop criteria*

Individual wishing to leave the study for any reason whatsoever will leave the study protocol, as will patients who cannot be satisfactorily managed within the study protocol (lack of therapeutic success, excessive side-effects, major surgical complications). The reasons for leaving the study will be documented, and patient outcome will continue to be documented (i.e. for intention-to-treat analysis).

7. Ethics

*7.1. Ethical conduct of trial*

The study will conform to the Geneva and Helsinki declarations and will be conducted according to GCP guidelines. The study design will be approved by the appropriate regional ethics committee.

*7.2. Subject information and consent*

Initial contact for recruitment will be via the oncological surgeon (in the surgical outpatient clinic, mammacare). At this time the patient will receive an information sheet and be warned of the possibility of a phone call about study participation. After a reflection time of at least 24 hours, patients will be contacted by phone to obtain preliminary verbal consent. At the time of the subsequent anaesthesia outpatient clinic visit (for premedication) the anaesthesiologist and research nurse will answer any further questions and obtain formal, written consent from the patient. The study nurse will at this time perform the necessary demonstration and training necessary for quantitative sensory testing. A copy of the patient information and consent form is attached as Appendix 8.1.

*7.3. Study drugs*

The safety record of COX-2 selective inhibitors is well proven, both acutely and chronically. Parecoxib is approved for perioperative acute use in the Netherlands in the dosage proposed (2X40mg); celecoxib is also approved in the Netherlands for chronic analgesic use at the proposed doses (2 X 200 mg per day).

## 8. References

Bar-Yosef S, Melamed R, Page GG, Shakhar G, Shakhar K, Ben-Eliyahu S. Attenuation of the tumor-promoting effect of surgery by spinal blockade in rats. Anesthesiology. 2001;94:1066-73

Barton SF, Langeland FF, Snabes MC, LeComte D, Kuss ME, Dhadda SS, Hubbard RC. Efficacy and safety of intravenous parecoxib sodium in relieving acute postoperative pain following gynecologic laparotomy surgery. Anesthesiology. 2002;97:306-14

Buckenmaier CC 3rd, Klein SM, Nielsen KC, Steele SM. Continuous paravertebral catheter and outpatient infusion for breast surgery. Anesth Analg. 2003;97:715-7.

Buvanendran A, Kroin JS, Tuman KJ, Lubenow TR, Elmofty D, Moric M, Rosenberg AG. Effects of perioperative administration of a selective cyclooxygenase 2 inhibitor on pain management and recovery of function after knee replacement: a randomized controlled trial. JAMA. 2003;290:2411-8.

Carpenter JS, Andrykowski MA, Sloan P, Cunningham L, Cordova MJ, Studts JL, McGrath PC, Sloan D, Kenady DE. Postmastectomy/postlumpectomy pain in breast cancer survivors. J Clin Epidemiol. 1998;51:1285-92

Chen LC, Elliott RA, Ashcroft DM. Systematic review of the analgesic efficacy and tolerability of COX-2 inhibitors in post-operative pain control. J Clin Pharm Ther. 2004;29:215-29.

Coderre TJ, Katz J, Vaccarino AL, Melzack R: Contribution of central neuroplasticity to pathological pain: review of clinical and experimental evidence. Pain 1993; 77:362-79

Dworkin RH, Hartstein G, Rosner HL, Walther RR, Sweeney EW, Brand L. A high-risk method for studying psychosocial antecedents of chronic pain: the prospective investigation of herpes zoster. J Abnorm Psychol. 1992;101:200-5

Gimbel JS, Brugger A, Zhao W, Verburg KM, Geis GS. Efficacy and tolerability of celecoxib versus hydrocodone/acetaminophen in the treatment of pain after ambulatory orthopedic surgery in adults. Clin Ther. 2001;23:228-41

Gottrup G, Andersen J, Arendt-Nielsen L, Jensen TS. Psychophysical examination of patients with post-mastectomy pain. Pain 2000;87:275-84

Greengrass R, Buckenmaier CC 3rd. Paravertebral anaesthesia/analgesia for ambulatory surgery. Best Pract Res Clin Anaesthesiol. 2002;16:271-83.

Kairaluoma PM, Bachmann MS, Korpinen AK, Rosenberg PH, Pere PJ. Single-injection paravertebral block before general anesthesia enhances analgesia after breast cancer surgery with and without associated lymph node biopsy. Anesth Analg. 2004;99:1837-43.

Kalso E, Perttunen K, Kaasinen S. Pain after thoracic surgery. Acta Anaesthesiol Scand. 1992;36:96-100

Karim A, Laurent A, Slater ME, Kuss ME, Qian J, Crosby-Sessoms SL, Hubbard RC. A pharmacokinetic study of intramuscular (i.m.) parecoxib sodium in normal subjects. J Clin Pharmacol. 2001;41:1111-9

Karmakar MK, Ho AM, Law BK, Wong AS, Shafer SL, Gin T. Arterial and venous pharmacokinetics of ropivacaine with and without epinephrine after thoracic paravertebral block. Anesthesiology. 2005;103:704-11.

Karmakar MK. Thoracic paravertebral block. Anesthesiology. 2001;95:771-80

Katz J, Jackson M, Kavanagh BP, Sandler AN. Acute pain after thoracic surgery predicts long-term post-thoracotomy pain. Clin J Pain. 1996;12:50-5

Kehlet H: Postoperative pain relief – what is the issue? Br J Anaesth 1994; 72:375-8

Liebeskind JC. Pain can kill. Pain. 1991;44:3-4

Ma W, Du W, Eisenach JC. Role for both spinal cord COX-1 and COX-2 in maintenance of mechanical hypersensitivity following peripheral nerve injury. Brain Res 2002;937:94-9.

McQuay HJ: Pre-emptive analgesia: a systematic review of clinical studies. Ann Med 1995; 27:249-56

Perkins FM, Kehlet H. Chronic pain as an outcome of surgery. A review of predictive factors. Anesthesiology. 2000;93:1123-33

Rasmussen GL, Steckner K, Hogue C, Torri S, Hubbard RC. Intravenous parecoxib sodium for acute pain after orthopedic knee surgery. Am J Orthop. 2002;31:336-43

Rodgers A, Walker N, Schug S, McKee A, Kehlet H, van Zundert A, Sage D, Futter M, Saville G, Clark T, MacMahon S. Reduction of postoperative mortality and morbidity with epidural or spinal anaesthesia: results from overview of randomised trials. BMJ. 2000;321:1493

Samad TA, Moore KA, Sapirstein A, Billet S, Allchorne A, Poole S, Bonventre JV, Woolf CJ. Interleukin-1beta-mediated induction of Cox-2 in the CNS contributes to inflammatory pain hypersensitivity. Nature 2001;410:425-427

Samad TA, Sapirstein A, Woolf CJ. Prostanoids and pain: unraveling mechanisms and revealing therapeutic targets. Trends Mol Med 2002;8:390-6

Sinatra R. Role of COX-2 inhibitors in the evolution of acute pain management. J Pain Symptom Manage. 2002;24(1 Suppl):S18-27. Review.

Straube S, Derry S, McQuay HJ, Moore RA. Effect of preoperative Cox-II-selective NSAIDs (coxibs) on postoperative outcomes: a systematic review of randomized studies. Acta Anaesthesiol Scand. 2005;49:601-13

Svensson CI, Yaksh TL. The spinal phospholipase-cyclooxygenase-prostanoid cascade in nociceptive processing. Annu Rev Pharmacol Toxicol 2002;42:553-83

Tasmuth T, Blomqvist C, Kalso E. Chronic post-treatment symptoms in patients with breast cancer operated in different surgical units. Eur J Surg Oncol. 1999;25:38-43

Tasmuth T, Kataja M, Blomqvist C, von Smitten K, Kalso E. Treatment-related factors predisposing to chronic pain in patients with breast cancer--a multivariate approach. Acta Oncol. 1997;36:625-30

Urban MO, Gebhart GF: Central mechanisms in pain. Med Clin North Am 1999; 83:585-96

Wall PD: The prevention of postoperative pain. Pain 1988; 33:289-90

Wallace MS, Wallace AM, Lee J, Dobke MK. Pain after breast surgery: a survey of 282 women. Pain. 1996;66:195-205

Wieseler-Frank J, Maier SF, Watkins LR. Glial activation and pathological pain. Neurochem Int. 2004;45:389-95.

Wilder-Smith OH, Tassonyi E, Senly C, Otten P, Arendt-Nielsen L. Surgical pain is followed not only by spinal sensitization but also by supraspinal antinociception. Br J Anaesth 1996;76:816-21

Wilder-Smith OH, Arendt-Nielsen L, Gaumann D, Tassonyi E, Rifat. Sensory changes and pain after abdominal hysterectomy: a comparison of anesthetic supplementation with fentanyl versus magnesium or ketamine. Anesth Analg 1998;86:95-101

Wilder-Smith OH: Pre-emptive analgesia and surgical pain. Progr Brain Res 2000;129:505-24

Wilder-Smith OHG, Tassonyi E, Arendt-Nielsen L. Preoperative back pain is associated with diverse manifestations of central neuroplasticity. Pain 2002;97:189-94

Wilder-Smith OHG, Tassonyi E, Crul BJP, Arendt-Nielsen L. Quantitative sensory testing and human surgery: Effects of analgesic management on postoperative neuroplasticity. Anesthesiology 2003,98:1214-22

Woolf CJ, Chong MS: Pre-emptive analgesia: treating postoperative pain by preventing the establishment of central sensitisation. Anesth Analg 1993; 77:362-79

Woolf CJ, Salter MW: Neuronal plasticity: increasing the gain in pain. Science 2000; 288:1765-8

Richmond CE, Bromley LM, Woolf CJ: Preoperative morphine pre-empts postoperative pain. Lancet 1993; 342:73-5

9. Appendices


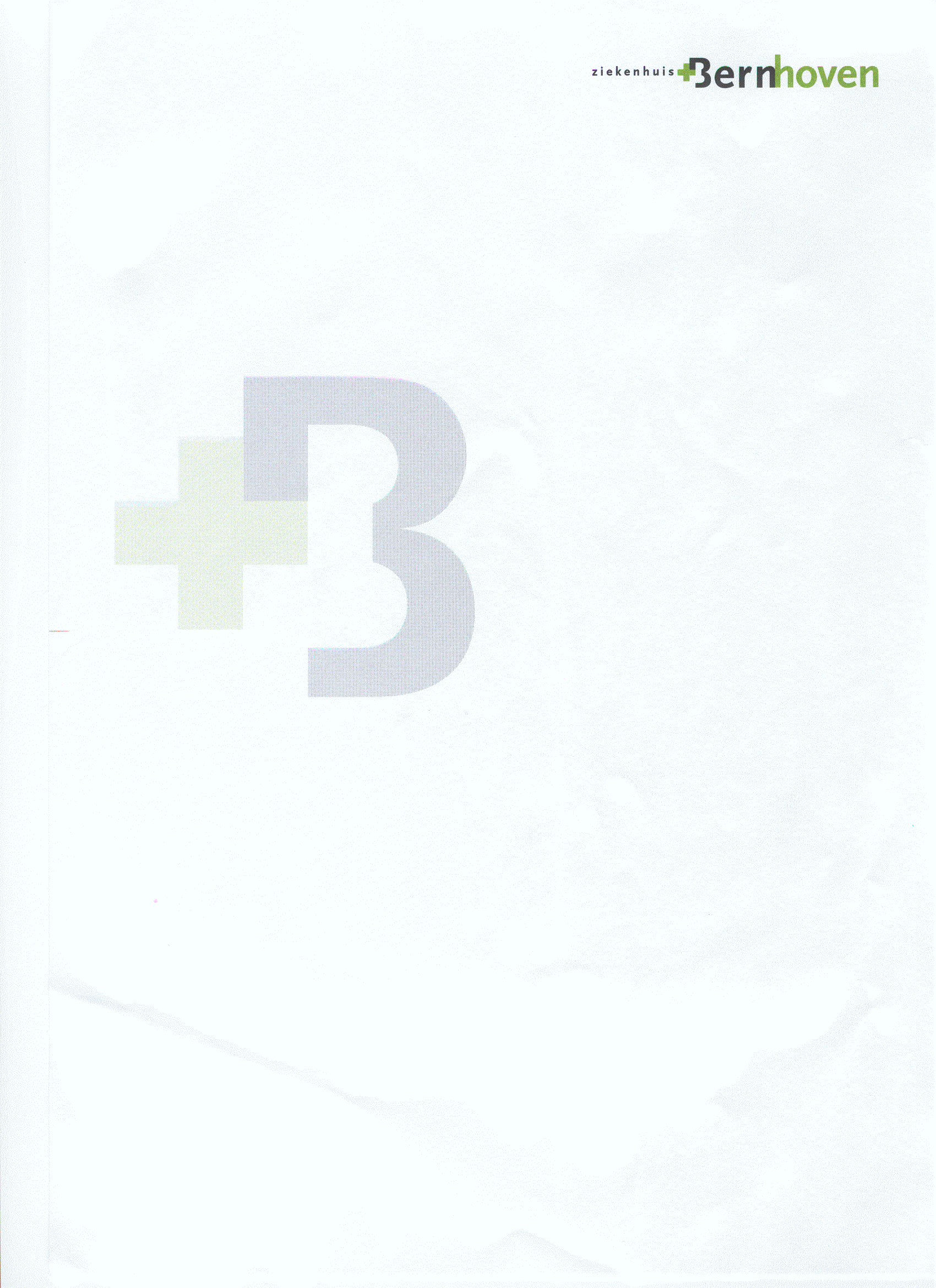
*9.1. Subject information and consent form*

Relative Contributions of Humoral and Nerve Nociceptive Inputs to Postoperative Central Sensitisation and Pain

Chronische pijn na een operatie aan de borst

# Patiënteninformatie


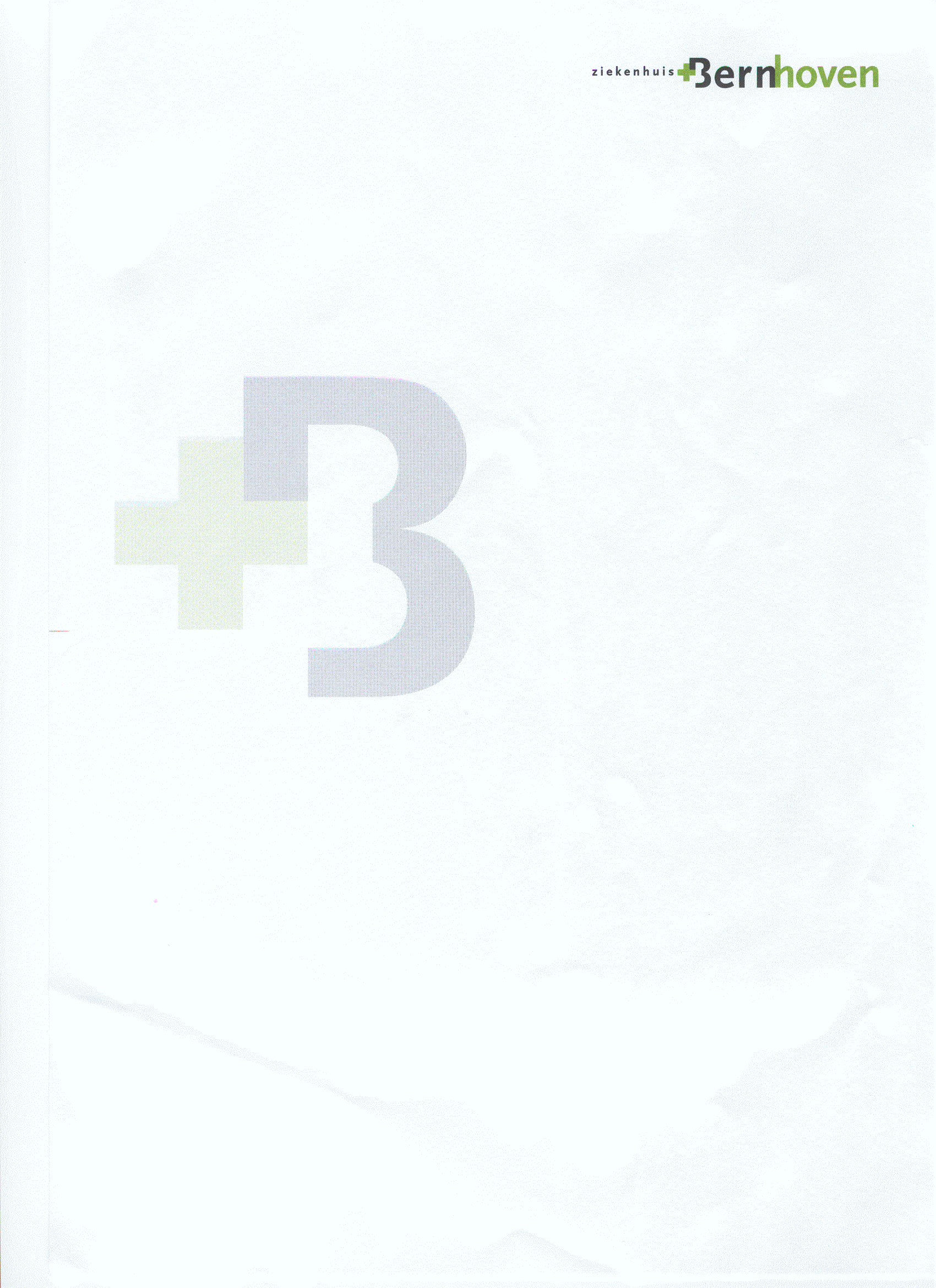
**Geachte mevrouw,**

Binnenkort zult u een operatie ondergaan aan uw borst en eventueel de lymfeklieren in uw oksel. Deze operatie is helaas noodzakelijk vanwege afwijkingen die in uw borst zijn gevonden. Graag willen wij uw aandacht vragen voor een wetenschappelijk onderzoek dat momenteel in dit ziekenhuis op de afdeling Anesthesiologie loopt.

**Voorkomen van chronische pijn**

Uit recent onderzoek is gebleken dat een hoog percentage van de vrouwen die een borstoperatie ondergaat op de lange termijn pijnklachten blijft houden. Deze chronische pijn is vaak moeilijk te behandelen. Wij zijn daarom op zoek naar methoden om het ontstaan van de chronische pijn te voorkomen. Het is nog onduidelijk hoe chronische pijn precies ontstaat, maar er zijn aanwijzingen dat een intensieve bestrijding van de acute pijn voor, tijdens en na de operatie het ontstaan van chronische pijn kan voorkomen.

**Plaatselijke verdoving**

Dit onderzoek houdt in dat de operatie bij u niet alleen onder algehele narcose zal worden uitgevoerd, maar dat er ook nog plaatselijke verdoving zal worden toegepast. Bij deze zogenaamde regionale anesthesie wordt dat gedeelte van uw lichaam waaraan u wordt geopereerd gevoelloos gemaakt voor pijnprikkels. Dit gebeurt door een injectie naast de wervelkolom aan de kant waar u wordt geopereerd. Deze techniek heeft als voordeel dat er nog urenlang na afloop van de operatie een pijnstillende werking zal optreden.

**Opzet van het onderzoek**

Verder zal onderzoek gedaan worden naar het effect van COX-2 remmer, een gangbare pijnstiller tegen acute pijn. De patiënten zullen in twee groepen worden verdeeld. De ene groep zal kort voor en zes uur na de operatie de COX-2 remmer toegediend krijgen, terwijl de andere groep dan een placebo of niet werkzame stof toegediend zal krijgen. Dit zal via het infuus gebeuren. Vervolgens zal u tot de ochtend van de vijfde dag tweemaal daags een tablet COX-2 remmer of een placebotablet voorgeschreven krijgen. Zowel de onderzoekers als uzelf zullen niet weten in welke groep u ingedeeld bent, wat betekent dat het gaat om een dubbelblind onderzoek.

**Wat gaat er verder gebeuren?**

Voor de operatie en 1, 5, 15 dagen en 1, 3, 6 en 12 maanden na de operatie zullen we de volgende metingen bij u komen doen. Hiervoor kunnen we bij u thuis langskomen op een moment dat het u schikt.

1. Invullen van vragenlijsten
2. Bepaling pijnscores. Hoeveel pijn heeft u? Een schaal van 0 (geen pijn) tot 10 (ondraaglijke pijn)
3. Afname van pijnvragenlijst. Hierbij wordt gebruik gemaakt van een lijst met 20 groepjes van woorden die gebruikt worden om pijn aan te duiden, bijvoorbeeld kloppend, bonzend, barstend etcetera.
4. Bepaling van de aanwezigheid van bijwerkingen aan de hand van bepaalde scoringstabellen.
5. Meten van uw pijngevoeligheid
6.
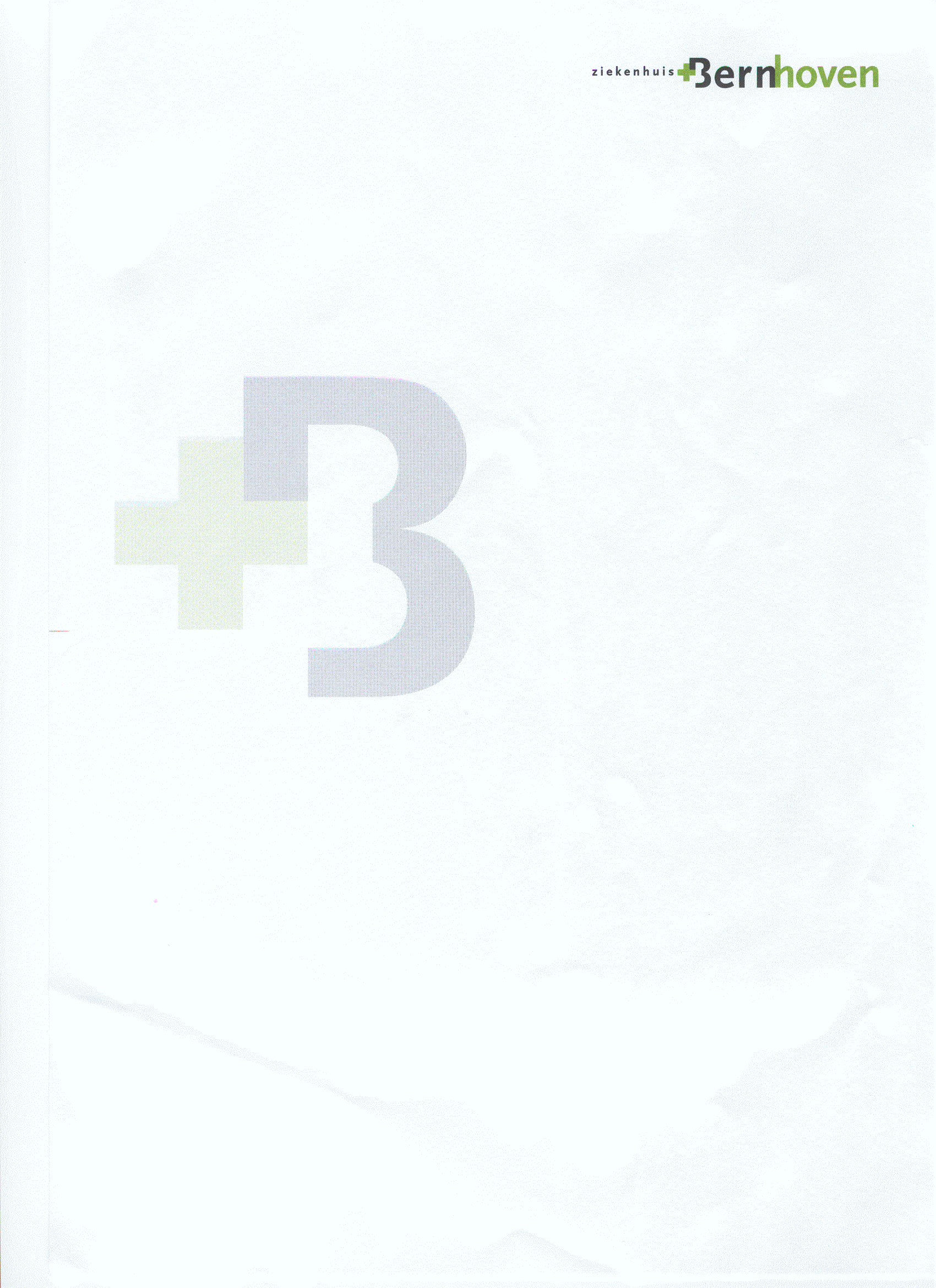
Ten eerste worden uw gevoel- en pijdrempels gemeten door middel van een apparaat dat pulsjes geeft op de huid via twee plakkers. U kunt te allen tijde de pulsjes zelf stoppen.
7. Met een kunststof draadje zal de huid rond het operatiegebied worden getest om te kijken of uw gevoel er veranderd is.
8. Voor de operatie wordt eenmalig de ijswatertest gedaan.
9. Uw pijndrempel wordt bepaald bij uw heup en pols door middel van druk.

Voor de eerste meting zult u een uitgebreide demonstratie krijgen van wat elke stap precies inhoud.

**Practische informatie**

Dit onderzoek wordt financieel gesteund door Pfizer, de producent van de COX-2 remmers Dynastat en Celebrex. De artsen die meedoen aan dit onderzoek zijn onafhankelijk en hebben geen belangen in de producent van de in dit onderzoek gebruikte medicatie. Verder zal er voor u een verzekering worden afgesloten. Dit is een standaard verplichte procedure voor iedereen die meedoet aan een onderzoek.

**Meedoen of niet?**

Meedoen aan dit onderzoek biedt u niet alleen de mogelijkheid gebruik te maken van een nieuwe techniek en intensief contact te onderhouden over uw behandeling, maar bovendien kunt u iets betekenen voor het verbeteren van de behandeling van de vrouwen die nog na u zullen komen. U bent uiteraard volkomen vrij al dan niet mee te doen aan het onderzoek, en u kunt zich op elk moment zonder opgaaf van redenen terugtrekken. Uw beslissing wordt volledig gerespecteerd en heeft op geen enkele wijze consequenties voor uw behandeling. Indien u besluit niet mee te doen met het onderzoek zal uw behandeling verlopen zoals dat gangbaar is: zonder de extra regionale verdoving en de extra COX-2 remmer.

**Vragen?**

U kunt met uw vragen terecht bij de onderstaande onderzoekers of op de pre-operatieve poli bij de anesthesioloog. Drs. A.P. Wolff, pijnspecialist in het UMC St. Radboud treedt op als onafhankelijk arts en is ook niet direct betrokken bij het onderzoek. Voor vragen kunt u hem bereiken op telefoonnummer 024 3614576.

C. Slegers Anesthesioloog Bernhoven 0412 621036

M. Steegers Anesthesioloog UMC St. Radboud 024 3614576

O. Wilder Smith Anesthesioloog UMC St. Radboud 024 3614576

S. Rijpma Onderzoeker 06 47658356

D. Snik Onderzoeker 06 38500086

**
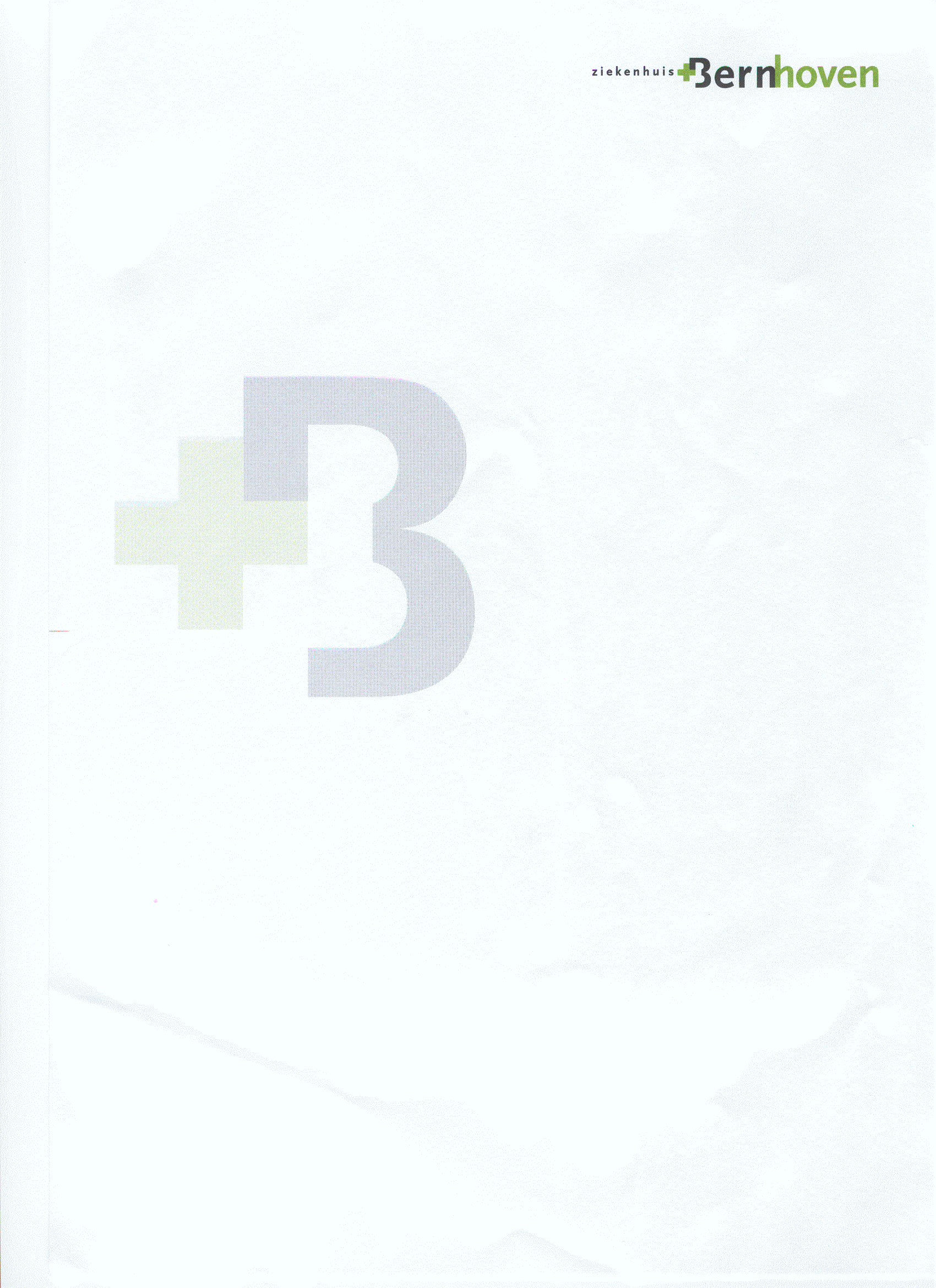
**

# **INFORMATIE PROEFPERSONENVERZEKERING**

Ziekenhuis Bernhoven heeft bij MediRisk, onderlinge waarborgmaatschappij voor instellingen in de gezondheidszorg, gevestigd te Utrecht, ten behoeve van deelnemers aan deze studie een verzekering afgesloten.

De verzekering voldoet aan de gestelde eisen voor medisch-wetenschappelijk onderzoek conform het Besluit van 23 juni 2003, inzake de verplichte verzekering bij medisch-wetenschappelijk onderzoek met mensen, in werking getreden met ingang van 1 september 2003.

## Verzekerde bedragen

A1 € 450.000,00 als maximum per aanspraak per proefpersoon, met een maximum van

€ 3.500.000,00 per afzonderlijk medisch-wetenschappelijk onderzoek,

met dien verstande dat indien de instelling meerdere wetenschappelijke onderzoeken verricht of heeft verricht het totale verzekerde bedrag is gelimiteerd tot € 5.000.000,00 voor schade die zich per verzekeringsjaar door medisch-wetenschappelijk onderzoek openbaart.

A2 Indien het wetenschappelijk onderzoek wordt uitgevoerd bij meerdere instellingen en de schade van de proefpersonen gedekt is door de verzekeringen van de instellingen waar zij aan het onderzoek deelnemen, is het bedrag waarvoor deze verzekeringen gezamenlijk voor dit onderzoek dekking verlenen € 3.500.000,00.

A3 De schade is gedekt indien deze zich heeft geopenbaard tijdens de deelname van de proefpersoon aan het onderzoek of binnen vier jaar na beëindiging van die deelname.

## Uitsluitingen ten aanzien van de proefpersonen

**De verzekering dekt niet:**

B1 Aanspraken voor schade die het gevolg is van het uitblijven van een vermindering van de gezondheidsproblemen van de proefpersoon, dan wel het gevolg is van de verdere verslechtering van de gezondheidsproblemen van de proefpersoon, indien de deelname van de proefpersoon aan het wetenschappelijk onderzoek geschiedt in het kader van de behandeling van deze gezondheidsproblemen.

B2 Aanspraken voor schade door aantasting van de gezondheid van de proefpersoon waarvan aannemelijk is dat die zich ook zou hebben geopenbaard wanneer de proefpersoon niet aan het onderzoek had deelgenomen.

B3 Aanspraken voor schade door aantasting van de gezondheid van de proefpersoon in het geval deze deelneemt aan een vergelijkend onderzoek als bedoeld in artikel 4 tweede lid van het Besluit verplichte verzekering en aannemelijk is dat de schade het gevolg is van de in dat lid bedoelde reeds toegepaste handeling waaraan de proefpersoon wordt onderworpen.

B4 Aanspraken voor schade die zich openbaart bij nakomelingen als gevolg van een nadelige inwerking van medisch-wetenschappelijk onderzoek op de proefpersoon en / of de nakomeling.

B5 Aanspraken voor schade waarvan het op grond van de aard van het medisch- wetenschappelijk onderzoek zeker of nagenoeg zeker is dat deze zich zou voordoen.


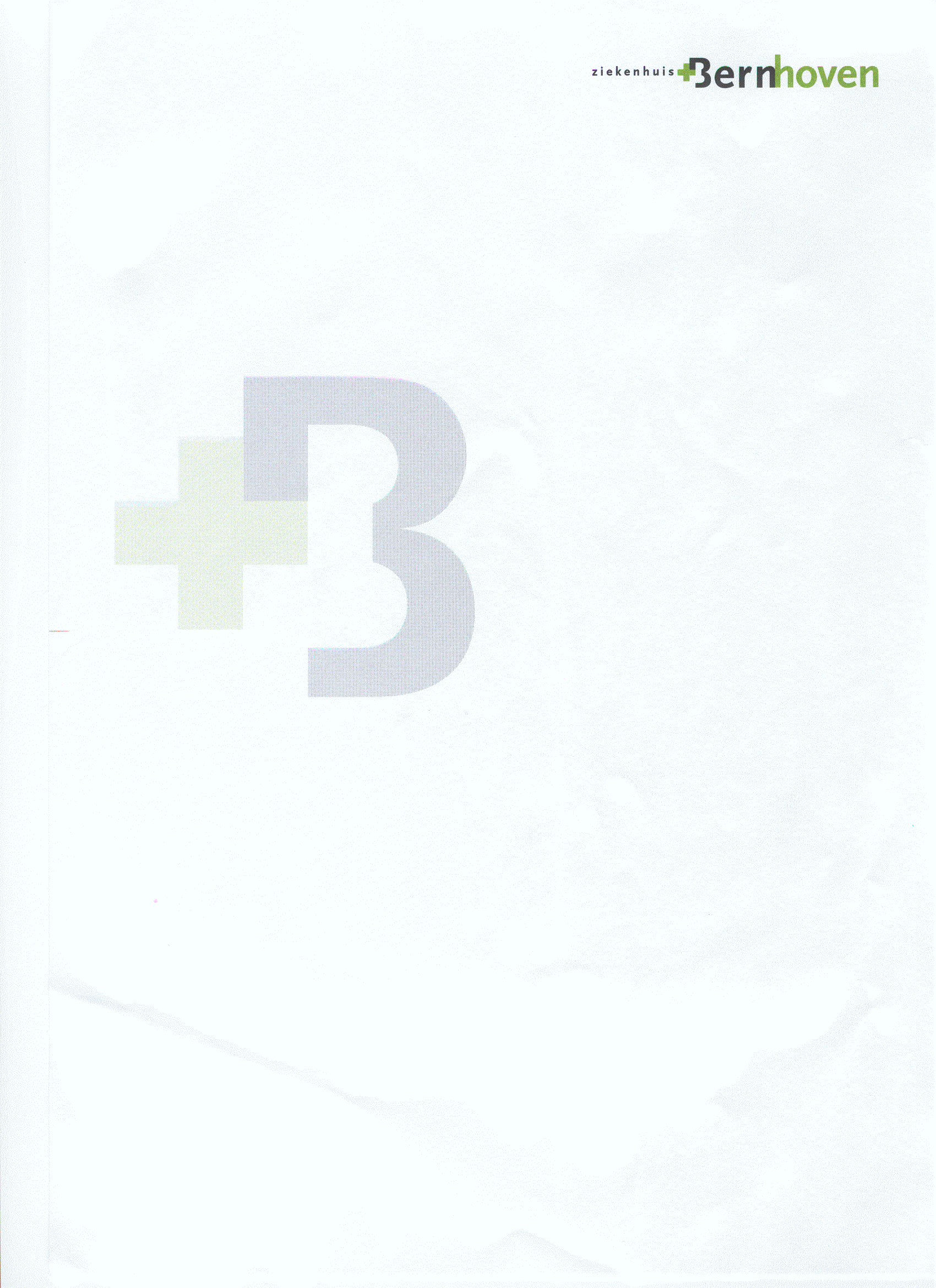
B6 Aanspraken voor schade die het gevolg is van het niet of niet volledig opvolgen van aanwijzingen en instructies door de proefpersoon, voor zover de proefpersoon daartoe in staat is.

**C. De verzekering dekt uitsluitend:**

C1 Schade geleden door de proefpersoon doordat deze verhinderd is arbeid te verrichten, tot een bedrag van ten hoogste € 60.000,00 per jaar.

C2 Schade door het derven van levensonderhoud geleden door de in artikel 108, tweede lid van Boek 6 van het Burgerlijk Wetboek genoemde personen tot in totaal een bedrag van ten hoogste € 60.000,00 per jaar.

C3 Kosten van huishoudelijke hulp voor een bedrag van ten hoogste € 7,50 per uur indien het inroepen van deze hulp redelijk is.

C4 Het recht op een vergoeding voor nadeel dat niet in vermogensschade bestaat, indien een vergoeding hiervan niet minder dan € 1.500,00 bedraagt, en voor zover het totaal van deze vergoedingen niet meer dan € 45.000,00 bedraagt.

C5 Schade als bedoeld in artikel 108, tweede lid, Boek 6 BW, tot een bedrag van ten hoogste

€ 10.000,00.

C6 Redelijke kosten van medische hulp, medische voorzieningen, hulpmiddelen en aanpassingen, tot een bedrag van ten hoogste € 50.000,00.

C7 Redelijke kosten van vervoer per taxi of per openbaar vervoer, en de redelijke kosten van eigen vervoer voor een bedrag van ten hoogste € 0,40 per kilometer, tot in totaal een bedrag van ten hoogste € 10.000,00.

## Verzekeraar

Onderlinge Waarborgmaatschappij voor Instellingen in de gezondheidszorg MediRisk B.A.

Atoomweg 100

Postbus 8409

3503 RK Utrecht

1. ^2^ [↑](#footnote-ref-2)
